# An exceptionally well-preserved monodominant fossil forest of *Wataria* from the lower Miocene of Japan

**DOI:** 10.1038/s41598-023-37211-z

**Published:** 2023-06-22

**Authors:** Megumi Nishino, Kazuo Terada, Kazuhiko Uemura, Yuki Ito, Toshihiro Yamada

**Affiliations:** 1 Botanical Gardens, Osaka Metropolitan University, Kisaichi, Katano, Osaka 576-0004 Japan; 2 Laboratory of Geology, Osaka Museum of Natural History, 1-23 Nagai Park, Higashi-Sumiyoshi-Ku, Osaka, 546-0034 Japan; 3 Fukui Prefectural Dinosaur Museum, Katsuyama, Fukui 911-8601 Japan; 4 Department of Geology and Paleontology, National Museum of Nature and Science, Tsukuba, Ibaraki 305-0005 Japan; 5 Department of Earth and Planetary Sciences, Faculty of Science, Hokkaido University, N10W8, Kita-Ku, Sapporo, 060-0810 Japan

**Keywords:** Plant evolution, Palaeontology

## Abstract

*Byttneriophyllum tiliifolium* is a leaf fossil-species of the family Malvaceae that was distributed widely throughout Eurasia from the Miocene to the Pliocene. An affinity to some Malvadendrina subfamilies has been suggested for *Byttneriophyllum*-bearing plants, but remains to be clarified due to insufficient information on other organs. Here, we report an exceptional lower Miocene fossil locality in Japan where a monodominant forest of the wood fossil-species *Wataria parvipora* flourished. Notably, the forest floor was covered by a bed consisting almost exclusively of *B. tiliifolium*. We observed occurrence modes of *B. tiliifolium* in this bed that confirmed that these leaves were deposited parautochthonously. These observations imply a biological connection between *B. tiliifolium* and *W. parvipora.* The wood and leaf characters together might narrow the affinity of *Byttneriophyllum*-bearing plants down to Helicterioideae within the Malvadendrina, although it is also possible that *Byttneriophyllum*-bearing plants constitutes an extinct lineage which is characterized by a combination of morphological traits found in several extant lineages. Our results suggest that *Byttneriophyllum*-bearing plants started to inhabit swamps no later than the end of the early Miocene when the global temperature was getting warmer.

## Introduction

Whole plants are rarely found as fossils because they are usually disarticulated into organs before burial^1–3^. On the other hand, extant plant taxa are usually defined by combinations of features found in various organs. Thus, reassembly of disarticulated organs into a whole plant is a critical step in efforts to understand the taxonomic affinity of a fossil plant^4^. Whole-plant reconstruction is preferably performed with articulated organs, but such finds are especially rare for large arborescent plants. The observation of spatiotemporal (dis)associations among organ occurrences is one way to “reassemble” disarticulated organs into a whole plant, as organs from the same plant likely occur together^3,4^. The accuracy of inference would likely be increased if we could determine associations among organs in fossil assemblages with fewer constituents, although the finding of an oligodominant assemblage is a matter of chance.

The extant mallow family (Malvaceae *s*.*l*.) is large, containing 249 genera that are distributed from the tropics to subtropics^5^. The distribution of this family was expanded to middle- to high-latitude areas during the warm periods of the Cenozoic^6^. *Byttneriophyllum tiliifolium* (A. Braun) Knobloch et Kvaček is a malvaceous leaf fossil-species that was distributed widely throughout Eurasia from the Miocene to the Pliocene^7–11^. However, its exact affinity remains to be established^11^ because leaf characters are not sufficiently informative to infer its taxonomic affinity within Malvaceae *s.l.*^12,13^. Thus, whole-plant reconstruction would be necessary to infer the infrafamilial affinity of *B*. *tiliifolium*.

**Figure 1:**
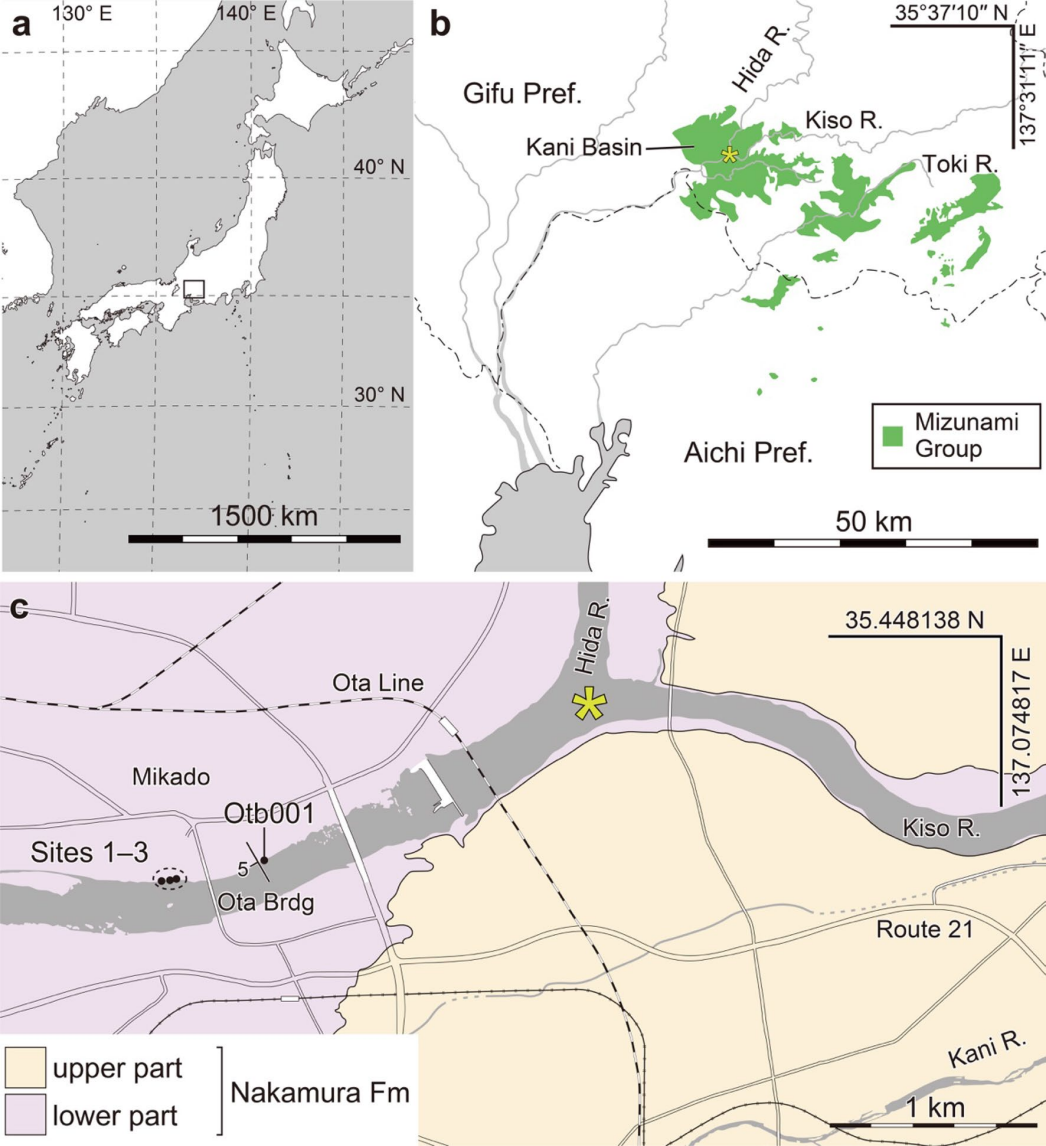
Study site locations and geology with base maps traced from the GSI maps^17^. (**a**) Locations of the study sites in Japan. The boxed area is shown close-up in panel (**b**). (**b**) Distribution of the Mizunami Group traced from Seamless digital geological map of Japan V2 1: 200,000^18^. The study sites are located in the Kani Basin. The position of the asterisk corresponds to that of the asterisk in panel (**c**). (**c**) Geological map of the study sites. See “Materials” for details on map sources.

**Figure 2:**
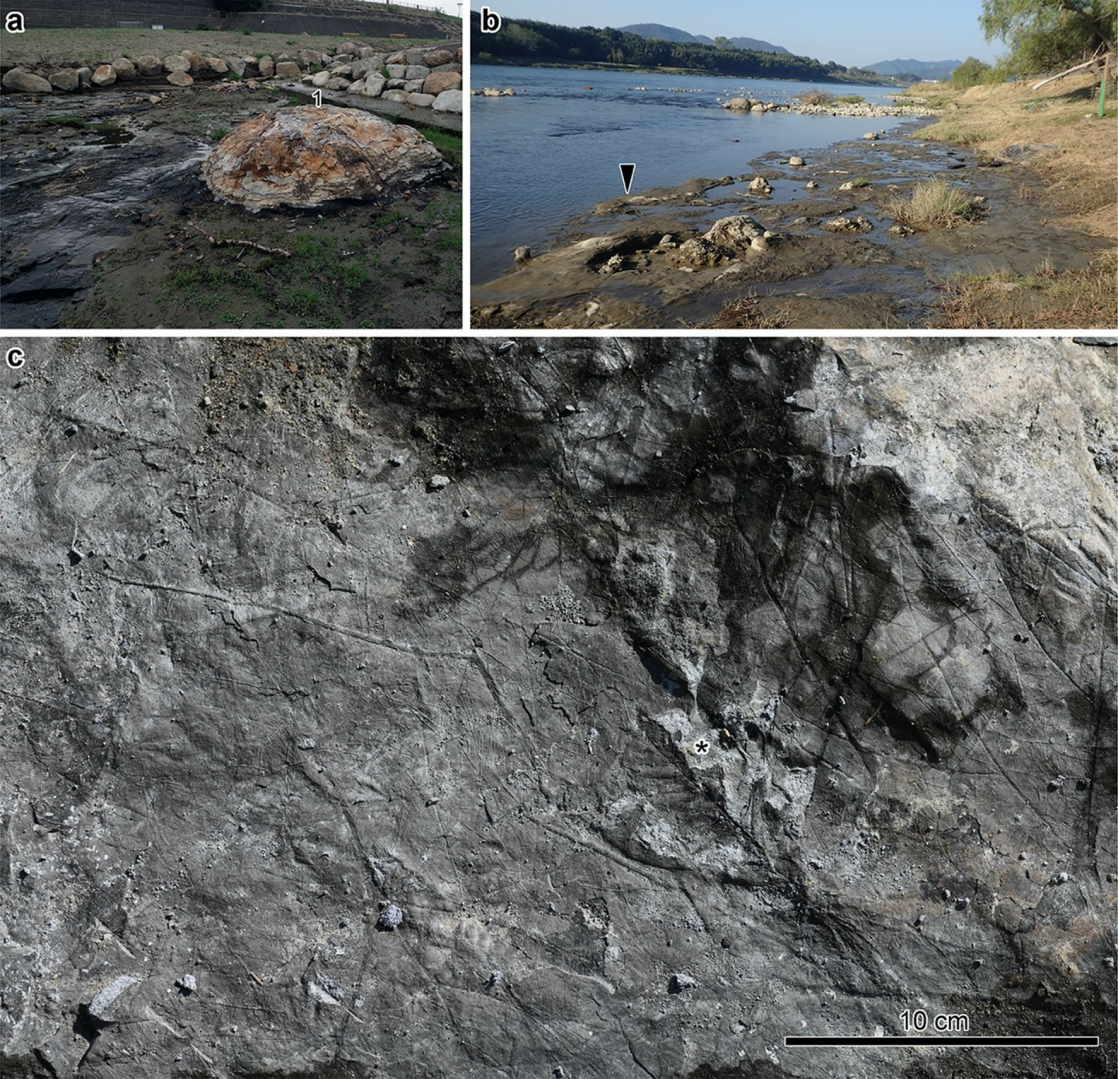
*Wataria* stumps and *Byttneriophyllum* bed in the PFP section. (**a**) Largest *Wataria* stump (individual #1, 137-cm trunk diameter) in the PFP section. (**b**) *Wataria* stumps found at site 2. The arrowhead shows the *Byttneriophyllum* bed from which leaf data were collected. (**c**) Surface views of the *Byttneriophyllum* bed at site 1. The surface was entirely covered by stacked *B*. *tiliifolium* leaves, except for one *Ulmus protojaponica* leaf (asterisk).

A fossil forest has been reported from the lower Miocene Nakamura Formation of the Mizunami Group, which is cropped out along the Kiso River, Minokamo City, Gifu Prefecture, central Japan (Figs. 1, 2)^14^. About 400 in situ stumps were found at the fossil site when an historic drought affected the Kiso River in 1994^14^; currently, most of these stumps are submerged. The composition of the fossil forest had not been clarified, as only 28 stumps had been examined taxonomically before the present study^15,16^.

In this study, we examined 137 stumps in a 2000-m^2^ area of the fossil site (Fig. 2a,b) and identified 130 stumps as *Wataria* K. Terada and M. Suzuki, a wood fossil-genus of Malvaceae *s.l*.^16^. We also found that the stumps were covered by a bed containing *B*. *tiliifolium* almost exclusively (Fig. 2c). We examined the occurrence modes of *B*. *tiliifolium* in the fossil forest, and showed that these leaves were deposited parautochthonously on the forest floor. These observations suggest that the fossil site represents a monodominant forest consisting of *Wataria* trees bearing *B. tiliifolium* leaves. We discuss the taxonomic and phytogeographic significance of these findings.

### Geological setting

The lower Miocene Mizunami Group consists of non-marine and marine sediments and is distributed in the Tono District of Gifu Prefecture and the Owari District of Aichi Prefecture, central Japan (Fig. 1a, b)^19^. In the Kani Basin, where the study sites are located, the Mizunami Group is composed only of non-marine sediments, divided in ascending order into the Hachiya, Nakamura, and Hiramaki Formations^14,20^. Fission track^20,21^, K-Ar^22^, and U-Pb^23^ methods consistently suggest ages of 22–19 Ma for the Hachiya Formation, 19 Ma for the Nakamura Formation, and 19–16 Ma for the Hiramaki Formation.

**Figure 3:**
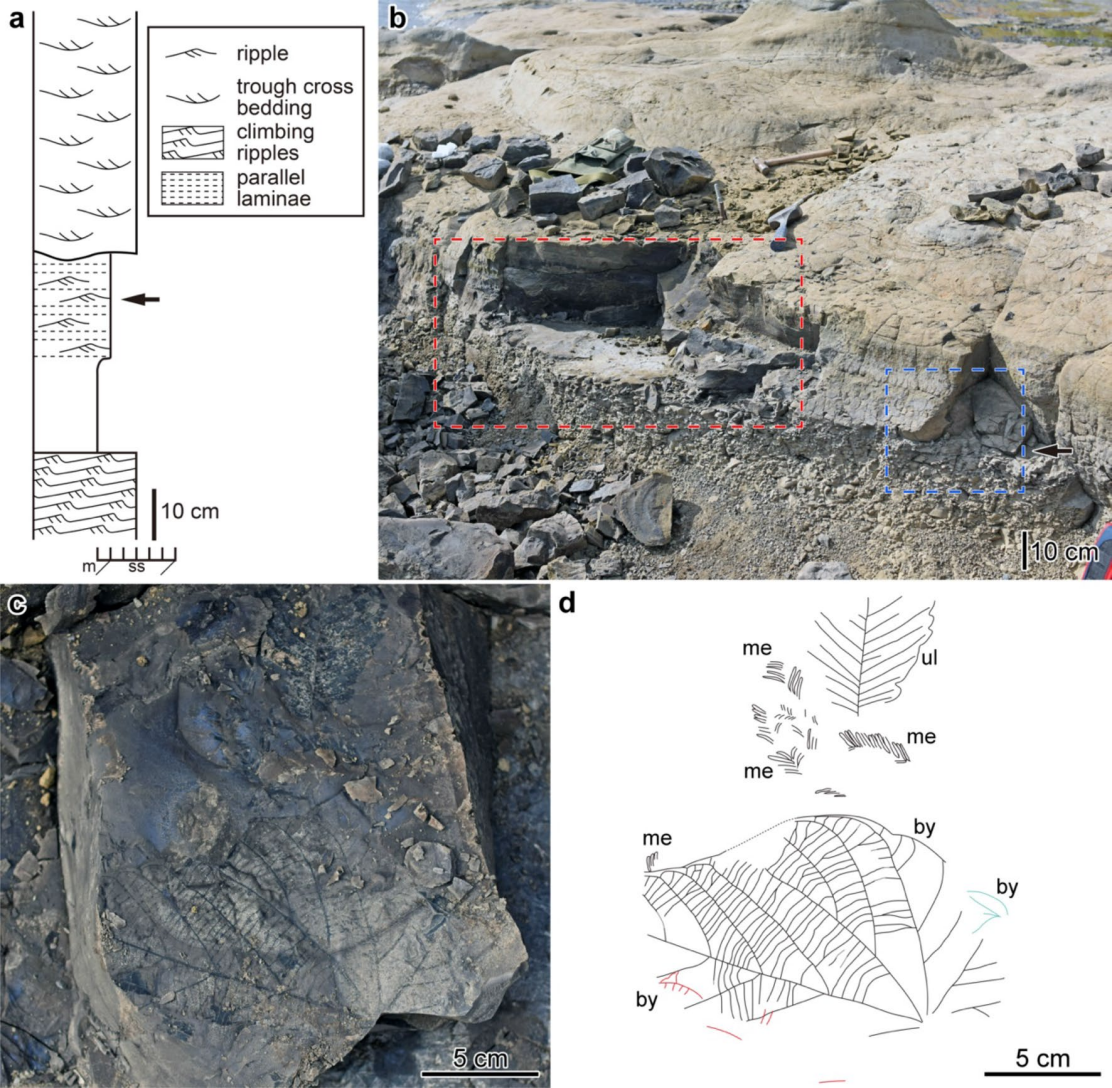
Lithology at locality Otb001. (**a**) Columnar section. (**b**) Photograph of the outcrop. Arrows in (**a**,**b**) indicate the plant-bearing bed. Red frame shows area where leaf occurrence data was taken. (**c**) Surface view of the plant-bearing bed observed in area of blue frame in (**b**). (**d**) Line drawing of the plant-bearing bed shown in (**c**). *Byttneriophyllum tiliifolium* (by) occurred with *Metasequoia occidentalis* (me) and *Ulmus protojaponica* (ul). Three *B. tiliifolium* leaves are piled which are traced by black, red, and blue lines, respectively.

**Figure 4:**
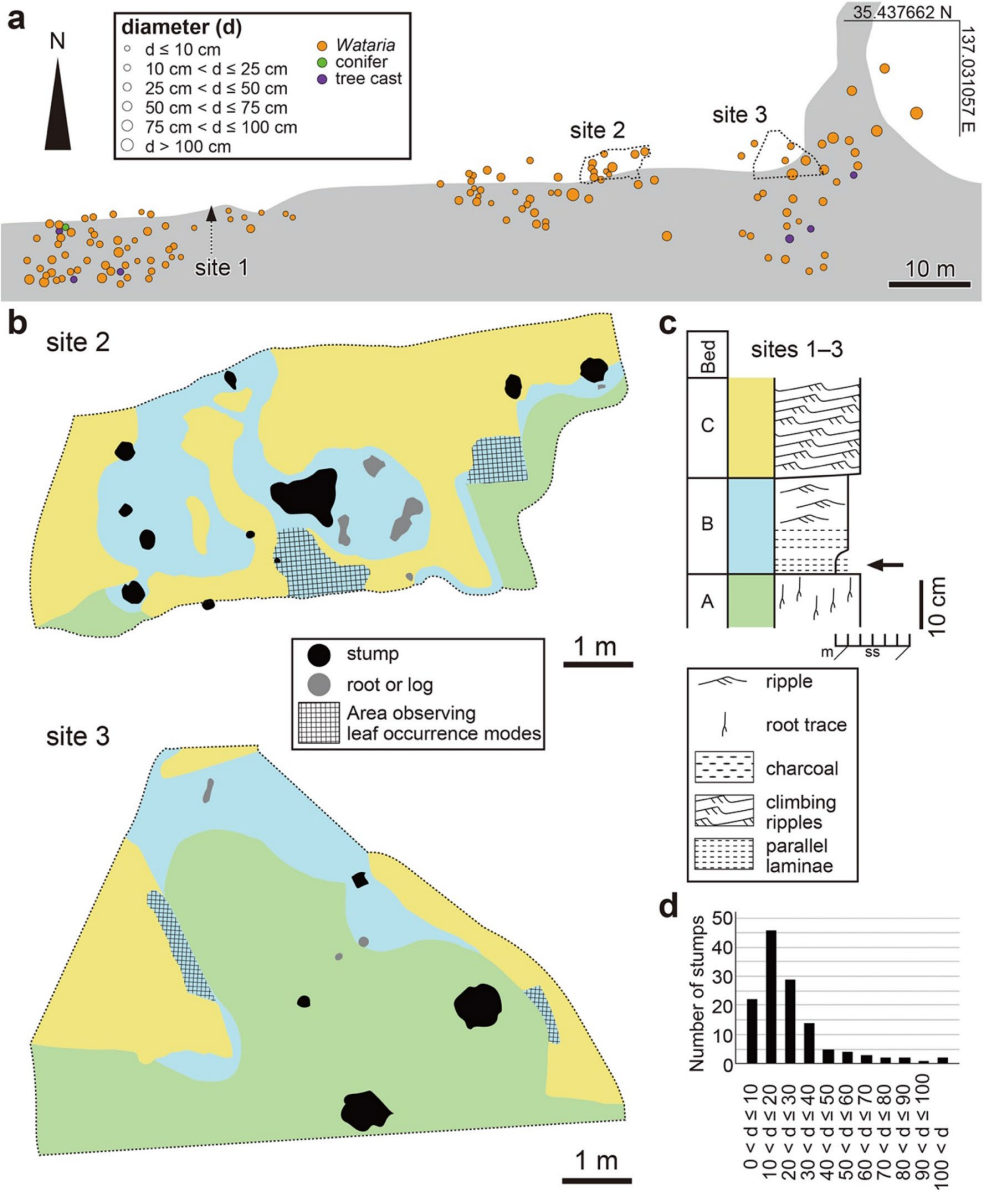
Distribution of in situ stumps and geology at sites 1–3. (**a**) Route map showing positions of in situ stumps. (**b**) Geological map of sites 2 and 3 showing surface exposure of beds A–C. The bed colors correspond to those in (**c**). (**c**) Columnar sections at sites 1–3. The arrow indicates the *Byttneriophyllum* bed. (**d**) Distribution of trunk diameters.

The study sites are located on the bed of the Kiso River near the Ota Bridge (Fig. 1c), where the fluvial siltstone and sandstone of the Nakamura Formation are exposed (Figs. 1, 2, 3, 4). The strata strike NW to NE and dip NW to SW or NE to SE by ≤ 5° (Fig. 1c). As the slope of the riverbed is almost parallel to the dip of the strata, almost the same horizon of the strata is exposed on the riverbed near the Ota Bridge. The in situ stumps are located mainly on the riverbed below the Ota Bridge in the Petrified Forest Park (PFP) of Minokamo City. Hereafter, we refer to the section below the Ota Bridge as the PFP section.

## Results

### Lithology of the study sites

At the Otb001 locality, upstream of the Ota Bridge, we observed a mudstone bed that had been eroded by overlying fine-grained sandstone with trough cross-beddings (Fig. 3a,b). The mudstone bed began with massive clay and transitioned to siltstone with ripple laminae, suggesting paleocurrents from NE to SW. *Byttneriophyllum tiliifolium* occurred with other leaves from the siltstone level (Fig. 3c,d). However, no stump was covered directly by leaf-bearing sediments.

The sediments cropped out in the PFP section (Figs. 2, 4a) consisted of three beds, one of which was exposed on the surface according to the extent of erosion. Bed A consisted of very fine-grained sandstone containing upright root traces (Fig. 4b,c). Bed B began with a laminated and carbonaceous mudstone layer containing a dense *B*. *tiliifolium* deposit. The grain size increased upward, and ripple laminae had developed in its upper part (Fig. 4b,c). Bed C was composed of very fine-grained sandstone with climbing ripples (Fig. 4b,c). Bed B represented mudstone deposited in a floodplain or back marsh, and beds A and C were crevasse splay flood deposits. The in situ stumps were anchored in bed A and the basal parts of the trunks were surrounded by bed B (Figs. 2, 4b,c).

### Distribution and taxonomical composition of in situ stumps

**Figure 5:**
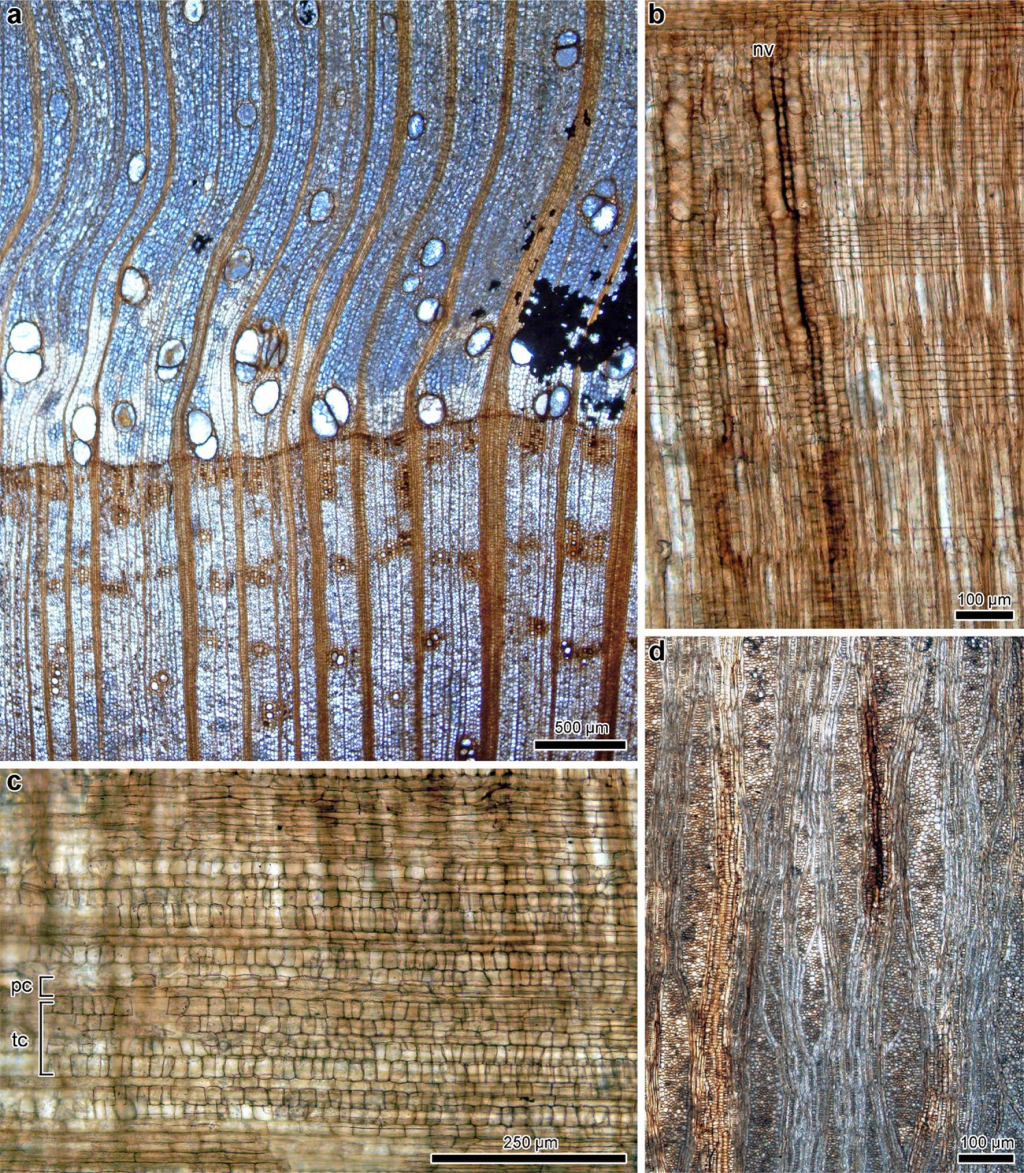
(**a–d**) *Wataria parvipora* from the Nakamura Formation in the PFP section. (**a**) Cross section showing parts of two annual rings with early wood in the center and late wood above and below. OSA-TB 9204-c1. (**b**) Multistoried axial parenchyma and narrow vessels (nv) in a radial section. OSA-TB 9204–r1. (**c**) Tile cells (tc) and procumbent cells (pc) in a radial section. OSA-TB 9204–r1. (**d**) Tangential section showing multiseriate rays and multistoried axial parenchyma. OSA-TB 9204–t1.

We found 137 in situ stumps in the PFP section (Fig. 4a,d, Supplementary Fig. 1). We measured the basal diameters of the trunks (Fig. 4d) and took thin sections for identification (Fig. 5, Supplementary Figs. 2–11). Most of the obtained wood fragments were somewhat deformed due to their close proximity to the roots. Some were diagenetically deteriorated (Supplementary Fig. 12). However, we identified 130 stumps as fossil-species of *Wataria* based on the following characters: tile cells of the *Pterospermum* type and intermediate *Durio* and *Pterospermum* types (sensu Chattaway^24^), multistoried axial parenchyma, and uniseriate or biseriate tangential bands of apotracheal parenchyma alternating with uniseriate to triseriate fiber rows (Fig. 5, Supplementary Figs. 2–11, Supplementary Note)^16^. Of these 130 stumps, 115 were assigned to *Wataria parvipora* Terada and Suzuki (Supplementary Table 1) because they had narrower early wood vessels than does *Wataria miocenica* and a narrower pore zone than does *Wataria oligocenica* (Supplementary Note)^16^. Fifteen of 130 *Wataria* stumps could not be identified at the fossil-species level because the vessels were highly deformed. One stump comprising seven non-*Wataria* stumps was identified as *Taxodioxylon* sp. This fossil-genus is recognized for its wood with distinct growth rings, abundant parenchyma, uniseriate rays, and the lack of resin canals (#37 in Supplementary Fig. 5)^25^. Six were mud casts in which no anatomically observable wood was preserved, so their taxonomic identities remained unclear (Fig. 4a).

The trunks of the largest and smallest *Wataria* stumps were 137 and 1 cm in diameter, respectively. The trunk diameters of 52% (*n* = 68) of the stumps were ≤ 20 cm (Fig. 4d). With the classification of trunk diameters in 10-cm increments, about one-third of the *Wataria* stumps belonged to the second smallest interval (10 cm < d ≤ 20 cm; Fig. 4d). The number of stumps almost halved per 10-cm increase between 10 and 50 cm (Fig. 4d). However, the decreasing trend was saturated for stumps with trunk diameters > 50 cm (Fig. 4d).

We found no clear relationship between the planar distribution and size of stumps, except that stumps with trunk diameters ≥ 75 cm were concentrated in the northeastern corner of the studied riverbed (Fig. 4a). However, we could not determine whether this distribution was statistically significant because these stumps were located on the margin of the exposed strata.

### *Byttneriophyllum* occurrence modes

**Figure 6:**
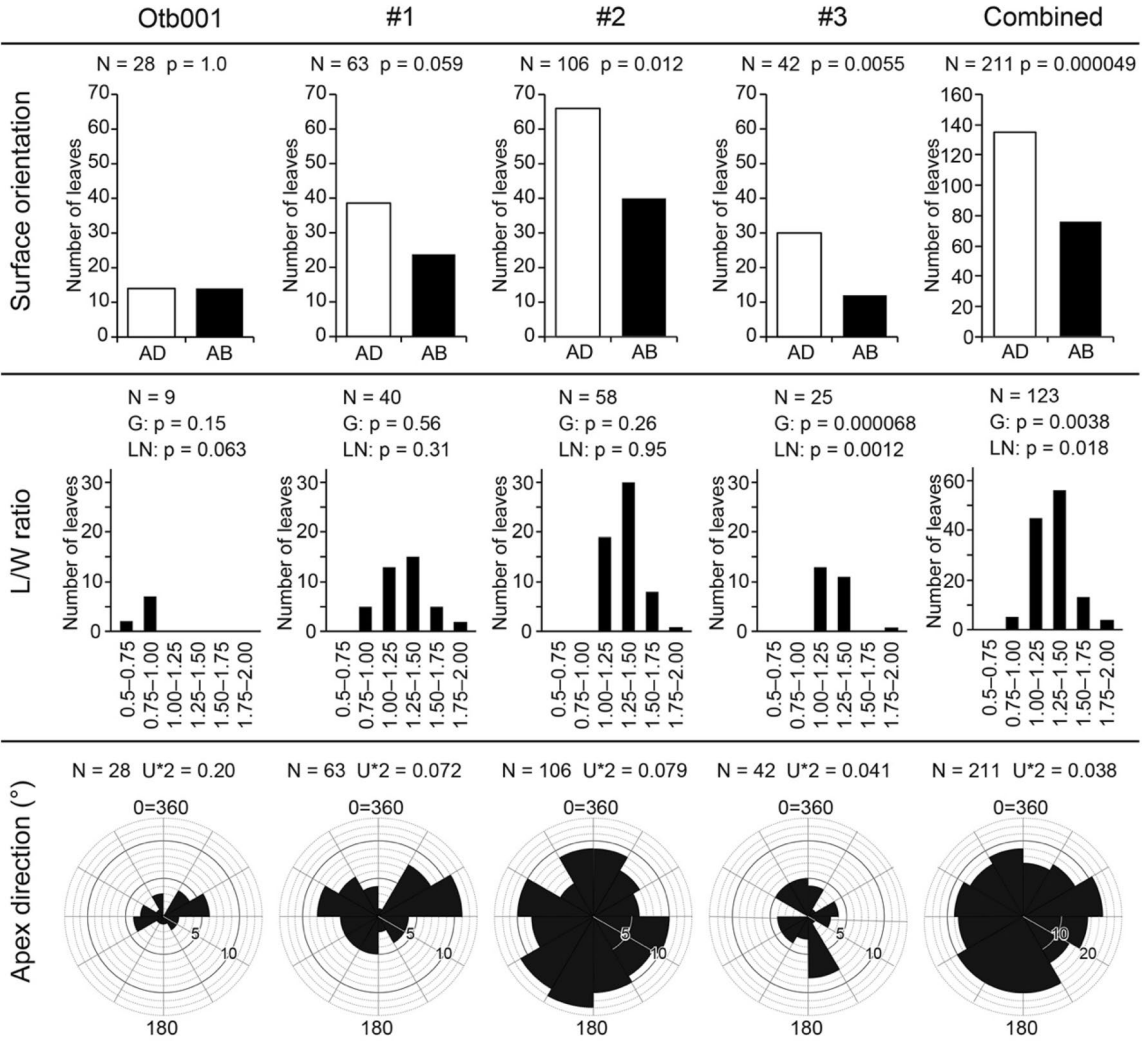
Occurrence modes of *Byttneriophyllum tiliifolium*. Numbers of leaves are given for the surface orientation, length-to-width (L/W) ratio, and apex orientation. Counts from sites 1–3 are summed in the combined column. The apex directions are shown in 360° with north set to 0° = 360°. *AB* abaxial upward, *AD* adaxial upward, *G* Gaussian distribution, *LN* log-normal distribution, *N* total number.

**Figure 7:**
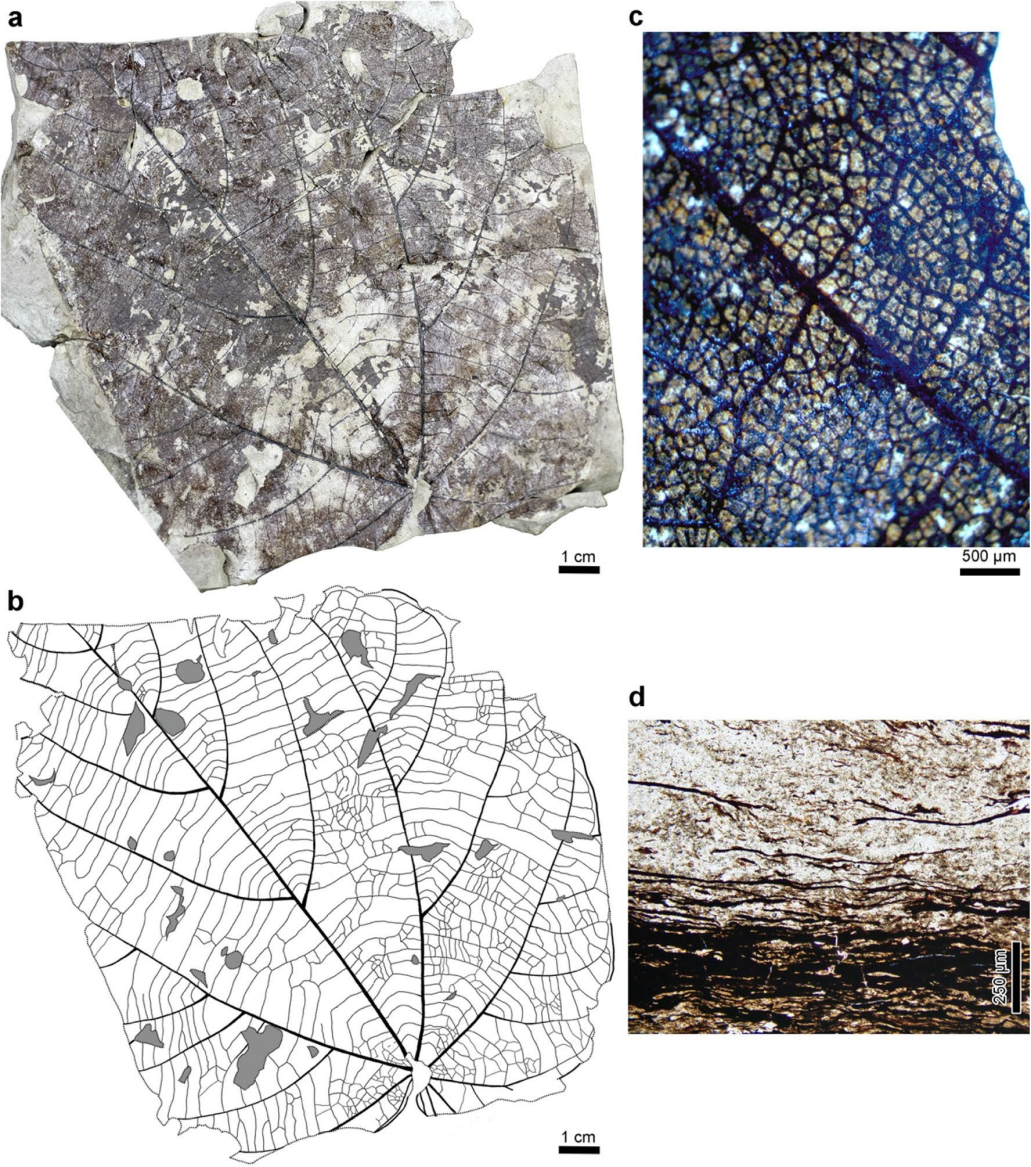
(**a–d**) *Byttneriophyllum tiliifolium* from the Nakamura Formation in the PFP section. (**a**) OSA-TB 9243–1 from site 3. (**b**) Line drawing of OSA-TB 9243–1. (**c**) Close-up of areoles in *B*. *tiliifolium* from site 3. OSA-TB 9243–3. (**d**) Vertical section showing dense deposits of *B. tiliifolium* leaves. OSA-TB 9244-1.

We collected data on the occurrence modes of *B. tiliifolium* from bed B at sites 1–3 and the Otb001 locality (Fig. 6). Locality Otb001 was chosen as a control site because no stump was found just below the leaf-bearing horizon. Leaves were identified based on shapes and venation patterns because epidermal characters could not be observed due to heavy coalification (Figs. 3c,d, 7a–c, Supplementary Note).

From a single bedding plane at locality Otb001 (Fig. 3a,b), we collected 28 leaves of *B*. *tiliifolium*, 4 leaves of *Ulmus protojaponica* Tanai et Onoe, and 2 leaves of *Metasequoia occidentalis* (Newb.) R.W. Chaney (i.e., 34 leaves in total; Fig. 3c,d). Thus, *B*. *tiliifolium* accounted for 82% of the collected leaves. Of the 28 leaves, 14 were buried with the adaxial side up and the remaining 14 were buried with the abaxial side up. No significant predominance was observed for leaf surface orientation during sedimentation (*p* = 1.0; Fig. 6). The distribution of length/width (L/W) ratios for nine leaves of *B*. *tiliifolium* did not deviate significantly from the Gaussian (*p* = 0.15) or log-normal (*p* = 0.06) distribution (Fig. 6). These leaves were buried with their long axes in the ENE to WSW directions (U*2 = 0.20; Fig. 6).

In bed B of the PFP section, *Byttneriophyllum* leaves (Fig. 7a–c) formed a pile ca. 1.5 mm thick (Fig. 7d). Quite thin clastic layers were intercalated between the mats of leaves in the part with the densest leaf concentration (Fig. 7d). We found 63 (98% of total leaves), 106 (98%), and 42 (95%) leaves of *B*. *tiliifolium* at sites 1–3, respectively, along with a few leaves of *U*. *protojaponica* (Fig. 2c). *Byttneriophyllum tiliifolium* tended to be found with the adaxial side up at sites 1–3 (Fig. 6). This tendency was statistically significant at sites 2 (*p* = 0.012) and 3 (*p* < 0.01), but not at site 1 (*p* = 0.059). At sites 1 and 2, the L/W ratios did not deviate significantly from the Gaussian (*p* = 0.56 and 0.26, respectively) or log-normal (*p* = 0.31 and 0.95, respectively) distribution. At site 3, the Gaussian (*p* < 0.001) and log-normal (*p* = 0.001) distributions of L/W ratios were not statistically supported (Fig. 6). No significantly predominant leaf apex direction was identified at any site (U*2 = 0.07, 0.08, and 0.04 at sites 1–3, respectively; Fig. 6).

As the leaf occurrence data from sites 1–3 were obtained from bed B, we also analyzed combined data from these three sites (*n* = 211 *B*. *tiliifolium* leaves in total). *Byttneriophyllum tiliifolium* represented 98% of the leaves obtained in this bed. Five *U*. *protojaponica* leaves obtained from this bed accounted for 2% of the total. Of the 211 leaves with observed dorsiventrality, 135 were buried with the adaxial side up (*p* < 0.001; Fig. 6). The L/W ratios, available for 123 leaves, deviated significantly from the log-normal (*p* = 0.02) and Gaussian (*p* < 0.01) distributions (Fig. 6). The apices of the 211 leaves exhibited no particular orientation (U*2 = 0.04; Fig. 6).

## Discussion

### Monodominant wood and leaf assemblages suggest a biological connection between *Wataria* and *Byttneriophyllum*

We found 130 *Wataria* stumps in the PFP section, which accounted for 95% of tree remains buried in the ca. 2000-m^2^ area (Fig. 4a). Other than *Wataria*, we found one *Taxodioxylon* stump and six tree casts of uncertain taxonomic identity. The percentage of *Wataria* was consistent with that observed in a previous study conducted in the PFP section [96% (27 of 28 stumps)]^15^. In addition, about half of the stumps were young trees with trunk diameters ≤ 20 cm (Fig. 4a,d), suggesting that the forest was repeatedly renewed by *Wataria*. These observations indicate that a *Wataria* monodominant forest flourished in the PFP section.

The *Wataria* stumps were anchored in bed A, which was overlain by bed B containing dense *B. tiliifolium* (Figs. 2, 4b,c). *Byttneriophyllum tiliifolium* accounted for ca. 98% of the total leaves obtained from bed B at sites 1–3 (Fig. 2c) and 82% of all leaves collected at the control Otb001 locality (Fig. 3c,d). Leaves that are highly represented in a leaf assemblage tend to have been shed from the parent trees, which grew close to the site of deposition^26,27^. Thus, the monodominance of *B*. *tiliifolium* suggests that trees bearing other leaves were quite rare in the forest of the PFP section.

In the part of bed B containing the greatest density of *Byttneriophyllum*, the leaves may have been deposited with limited transport by water because clastic particles were intercalated very thinly between them (Fig. 7d). This inference is also supported by the surface and apex orientations of the leaves. *Byttneriophyllum* apices were oriented in unspecified directions at sites 1–3, in contrast to the preferred NE or SW orientation at Otb001 (Fig. 6). The former observation suggests that *Byttneriophyllum* leaves were not mixed with water currents before burial at sites 1–3^26,27^, and the latter suggests that the leaves were oriented by NE to SW paleocurrents, which were dominantly observed at the Otb001 locality. *Byttneriophyllum* leaves tended to be deposited with the adaxial surface upward at sites 1–3 (Fig. 6), although this was not a significant pattern at site 1. This observation was in marked contrast to the equal numbers of adaxial-side-up and abaxial-side-up leaves at Otb001 (Fig. 6). Surface orientation preferences are determined by the aerodynamic conditions under which leaves are placed, but they become less obvious for waterlogged leaves^27^. Thus, the *Byttneriophyllum* layer in bed B likely represents leaf litter deposited parautochthonously at the feet of the parent trees.

Leaf L/W ratios in a species population likely have a Gaussian or log-normal distribution^27^. Thus, these ratios also have these distributions in a parautochthonous assemblage^27^. However, combined L/W ratios from sites 1–3 deviated from the Gaussian and log-normal distributions; such distributions were possible for data from sites 1 and 2 (Fig. 6). These observations could be explained in two ways: the distribution of L/W ratios actually deviated from the Gaussian or log-normal distribution in the original population, or defoliation occurred in a ratio-dependent manner. Larger datasets from various localities should be assembled to test these possibilities.

These data suggest based on their close association that the *Wataria* and *B. tiliifolium* constitute a whole plant. The deposits containing them show that a monodominant *Wataria*-*Byttneriophyllum* forest flourished in a swampy environment on a floodplain. Based on the frequent associations of *B*. *tiliifolium* with lignite layers, this fossil-species is assumed to constitute swampy vegetation occurring in Europe during the Miocene to Pliocene^7,10,11,28–31^. Our results support the inferences made from the European evidence. On the other hand, *Wataria* has been reported only from Asia^15,16,32–36^. The absence of *Wataria* records in Europe might imply that more than two fossil-species bore *B. tiliifolium*-type leaves. This possibility could be tested by finding leaf compressions from the Nakamura Formation which preserve epidermal features helpful for accurate identification of *B. tiliifolium*^11^. In addition, *Wataria* should be explored in European *B. tiliifolium* localities.

It is suggested that a whole plant bearing *B. tiliifolium* sheds fruit of *Banisteriaecarpum giganteum* (Göppert) Kräusel^37^ and pollen of *Intratriporopollenites instructus* (Potonié) Thomson et Pflug^11^ in Europe. We did not find *Ba. giganteum* at the study site (see below as well), and we have not conducted palynological analyses to search for *I. instructus*. The search for these fossil-species would also be helpful in evaluating the taxonomic relationship between European and Japanese *B. tiliifolium*.

### Possible affinity of *Byttneriophyllum*-bearing plants

Phylogenetic analyses have led to the identification of two major clades within Malvaceae *s*.*l*.: Byttneriina and Malvadendrina^6,13,38^. Byttneriina consists of Grewioideae and Byttnerioideae, and Malvadendrina has seven subfamilies (Bombacoideae, Brownlowioideae, Dombeyoideae, Helicterioideae, Malvoideae, Sterculioideae, and Tilioideae)^6,13^. The phylogenetic relationships among the Malvadendrina subfamilies remain to be established, but the Malvatheca clade, consisting of Bombacoideae and Malvoideae, is well supported by molecular phylogenetic analyses^6,38^.

*Byttneriophyllum tiliifolium* has been considered to be a leaf fossil-species of Malvaceae *s*.*l*.^11,37^, but its precise infrafamilial position could not be determined based on leaf characters alone^11^. However, it has stellate and multicellular clavate trichomes on the leaf epidermis, which are not found in Malvatheca^11^ but are found in some non-Malvatheca genera of Malvadendrina, such as *Brownlowia* (Brownlowioideae), *Firmiana*, and *Hildegardia* (Sterculioideae)^11^. Thus, *B*. *tiliifolium* may belong to a non-Malvatheca subfamily of Malvadendrina (Brownlowioideae, Dombeyoideae, Helicterioideae, Sterculioideae, or Tilioideae)^11^.

The fossil-genus *Wataria* is characterized by tile cells in layers that represent an intermediate type between the *Durio* and *Pterospermum*^16^ types sensu Chattaway^24^. The extant malvalean genera basically have the tile cells^39^, but the intermediate type is found in only four genera of the Malvaceae *s*.*l*. [*Grewia* (Grewioideae), *Guazuma* (Byttnerioideae), *Reevesia* (Helicterioideae), and *Triplochiton* (Helicterioideae)]^16,40^. Among these, only *Triplochiton* shares with *Wataria* axial xylem parenchyma characters such as a uniseriate or biseriate apotracheal parenchyma and uniseriate to triseriate vasicentric paratracheal parenchyma, implying that these genera are closely related^16^.

*Byttneriophyllum tiliifolium* leaves have been found to occur with samaras of *Banisteriaecarpum giganteum* at many localities in Europe^37^. Thus, a biological connection between *B. tiliifolium* and *Ba. giganteum* has also been suggested^37^. Although we did not find any fruit remains in the bed B at sites 1–3, *Ba. giganteum* samaras often occur with *B. tiliifolium* in the Nakamura Formation (Supplementary Fig. 14, Supplementary Note), supporting the biological connection between them. *Banisteriaecarpum* is similar to the samaras of *Heritiera* (Sterculioideae)^37,41^, *Mansonia* (Helicterioideae)^42^, and *Triplochiton*^43,44^.

In short, several infrafamilial affinities were inferred for each of *B*. *tiliifolium* leaves, *W*. *parvipora* woods, and *Ba. giganteum* samaras which possibly constitute a whole plant. These inferences could be consistent if the fossil-species bearing these organs belongs to the Helicterioideae. However, it is also possible that the fossil-species constitutes an extinct lineage characterized by a mosaic combination of morphological traits which are separately found in several extant lineages.

### Climatic implication of *Byttneriophyllum*-bearing plants

The spatiotemporal distributions of extant and fossil Malvaceae species suggest that they tend to favor tropical climates^5,6^. Consistent with this tendency, we have shown that *B*. *tiliifolium* began to inhabit swamps no later than the end of the early Miocene, when subtropical to warm temperate climates^45^ prevailed in mid-latitudinal areas^46–49^. *Wataria* were also found in the subtropical to warm temperate mid-latitudinal areas of Asia during the early Oligocene to middle Miocene^16,32–36^. *Triplochitioxylon oregonensis* Manchester, a possibly related to *Wataria*, was reported from the middle Eocene Clarno Formation in Oregon, USA which deposited in tropical to subtropical area^40,50^.

Global climate cooling began in the middle Miocene^51^, but temperature would be still warm enough for *B*. *tiliifolium* to thrive in the swamps of Europe during the later middle Miocene to the early Pliocene^9,10,28–31^. It has also been reported from the upper Miocene in Japan^8^. However, *B. tiliifolium*-bearing plants would not be able to survive much cooler conditions after the early Pliocene^51^.

## Methods

We examined the occurrence modes of *B. tiliifolium* in the bed of the Kiso River near Ota Bridge, Mikado, Minokamo City, Gifu Prefecture, where the middle part of the Nakamura Formation crops out (Fig. 1c)^14^. We observed bed B, which contained dense *B*. *tiliifolium* deposits and covered *Wataria* stumps, at three sites in the PFP section: site 1 (35° 26′ 17″ N, 137° 1′ 48″ E), site 2 (35° 26′ 15″ N, 137° 1′ 50″ E), and site 3 (35° 26′ 17″ N, 137° 1′ 51″ E). We also set one control site containing no *Wataria* stump in the upstream area of the Ota Bridge (Otb001 locality; 35° 26′ 17″ N, 137° 2′ 7″ E; Fig. 1c). We exposed a single plane of 0.7–1.6 m^2^ to observe the occurrence modes at each site, although the plane could include several lamina planes.

We collected data for the following indices^27^ at each site to identify whether *B*. *tiliifolium* leaves were trapped parautochthonously in the sediments: the occupancies of each component species, leaf surface orientations (adaxial-side-up or abaxial-side-up), L/W ratios, and leaf apex directions. The occupancies are proportions of *B*. *tiliifolium* in a leaf assemblage based on the number of leaves. In this study, leaf length was defined as the distance from the leaf apex to the point where the lamina attached to the petiole, and leaf width was defined at the widest transect of the leaf. The imaginary line corresponding to the leaf length nearly parallel to the midrib was used for the recording of the leaf apex direction. Angles from north were recorded in the range of 0° to 360°. The observed directions were plotted onto a rose diagram with 24 classes defined at 15° intervals.

The normality of the distributions of L/W ratios and log-transformed values was tested using the Shapiro–Wilk test. We adopted a significance level of *p* = 0.05 to reject the null hypothesis that size measurements were distributed normally. Leaf surface orientation preferences were analyzed using the chi-squared test with a significance level of *p* = 0.05. The goodness of fit of leaf direction data was assessed using Watson’s U^2^ test^52,53^ with the null hypothesis that the directions were distributed randomly. The significance level was set to *p* = 0.05, which yielded a U*2 value of 0.187. Thus, the null hypothesis was not rejected when the U*2 value was < 0.187.

The plant fossil collection and use was in accordance with all the relevant guidelines. M.N. made initial wood and leaf identifications, and K.T. and K.U. confirmed them. The collected specimens were deposited in the Tertiary Paleobotanical Collections of the Osaka Museum of Natural History, Osaka, Japan (OSA-TB), or in the Paleobotanical collections of the National Museum of Nature and Science, Tsukuba, Japan (NSM-PP) under following registration numbers: OSA-TB 9100, 9104–9244, NSM-PP-23947, 23949 (Supplementary Tables 1, 2).

We traced an index map (Fig. 1a) from a topographic map available on the “GSI Maps” website^17^, which was provided by the Geospatial Information Authority of Japan (GSI). To trace the distributions of the Mizunami Group (Fig. 1b), we used the “Seamless digital geological map of Japan V2 1: 200,000”^18^ provided by the Geological Survey of Japan (GSJ), the National Institute of Advanced Industrial Science and Technology, in combination with the GSI Maps^17^. To ensure accuracy, we compared our traced distribution with that presented by Itoigawa^19^. For the geological map of the study area (Fig. 1c), we overlaid our own geological observations onto GSI maps^17^, while adopting the names for geological units from Shikano^14^. We also verified that our own geological map is consistent with that of Shikano^14^.

## Supplementary Information

Supplementary Information.

## Data Availability

The datasets used and/or analysed during the current study available from the corresponding author on reasonable request.
