# Supplementary material for: An exceptionally well-preserved monodominant fossil forest of Wataria from the lower Miocene of Japan
Source: Sci Rep. 2023 Jun 22;13:10172. doi: 10.1038/s41598-023-37211-z (PMC10287665; doi:10.1038/s41598-023-37211-z)
Supplement: Supplementary file 1 — Supplementary Information. [file 41598_2023_37211_MOESM1_ESM.pdf]

Supplementary Information for

An exceptionally well-preserved monodominant fossil forest of *Wataria* from the lower Miocene of Japan

Megumi Nishino, Kazuo Terada, Kazuhiko Uemura, Yuki Ito, Toshihiro Yamada*

*To whom correspondence (ptilo@nifty.com)

This PDF file includes:

1. **Supplementary Figures (Figure 1–14)**
2. **Supplementary Notes**
3. **Supplementary Table 1**
4. **Supplementary Table 2**

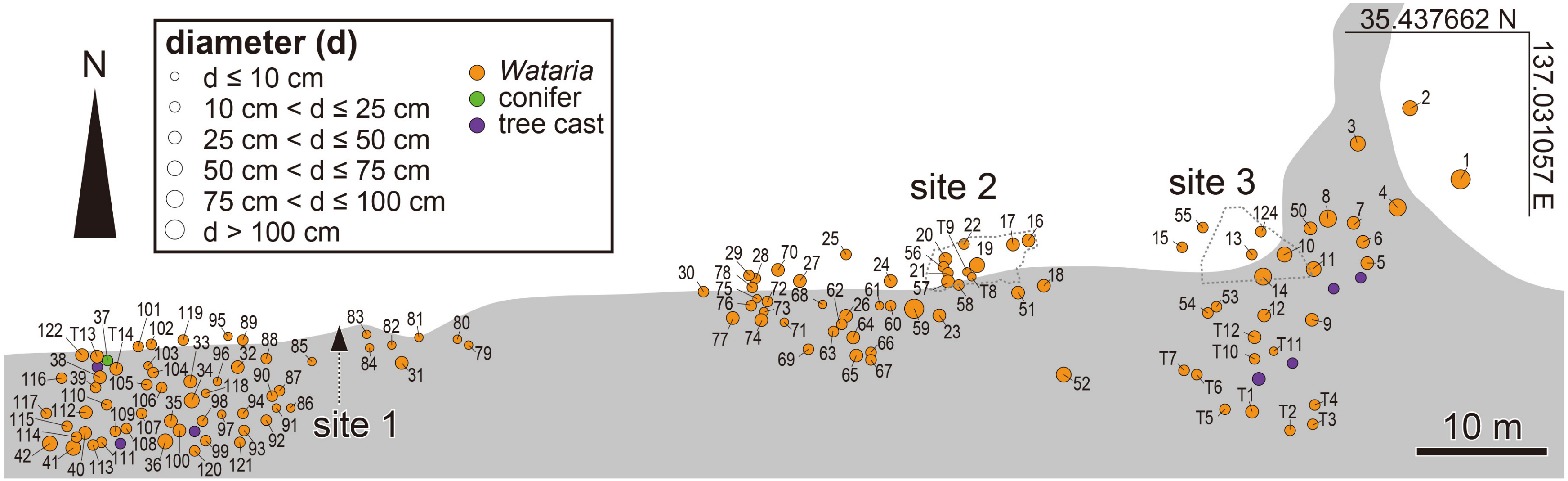

Supplementary Figure 1. Individual numbers assigned to *in situ* stumps.

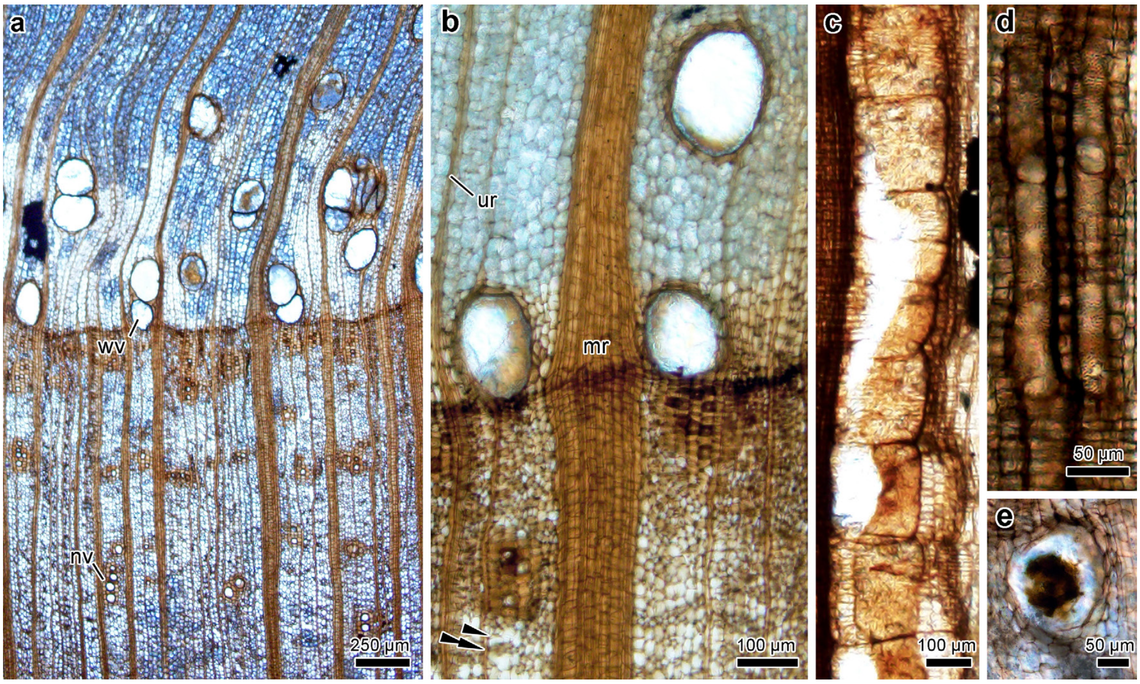

Supplementary Figure 2. *Wataria parvipora* from the Nakamura Formation in the PFP section (OSA-TB 9204, individual #101). (**a**) Cross-section. Early and late woods contain wide vessels (wv) and narrow vessels (nv), respectively. OSA-TB 9204–c1. (**b**) Close-up of cross-section. Note the alternating tangential bands of parenchyma cells (arrowhead) and fibers (double arrowhead). mr, multiseriate ray; ur, uniseriate ray. OSA-TB 9204–c1. (**c**) Wide vessel in radial section. OSA-TB 9204–r1. (**d**) Narrow vessels in radial section. OSA-TB 9204–r1. (**e**) Gum-like material in cross section of a wide vessel. OSA-TB 9204–c2.

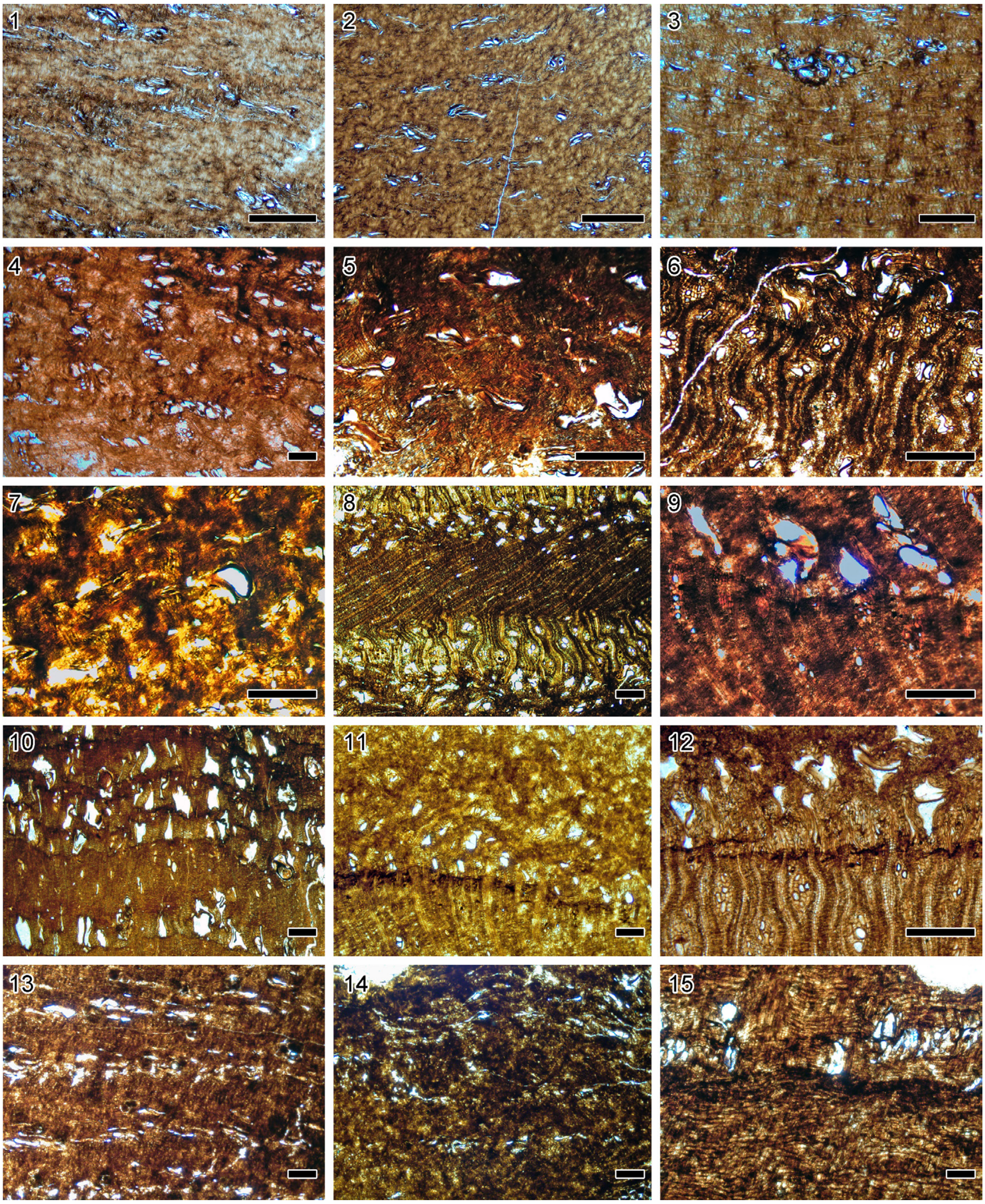

Supplementary Figure 3. *Wataria parvipora* from the Nakamura Formation in the PFP section. Numbers correspond to individual numbers in Supplementary Figure 1. Scale bars are 250 μm. Cross-sections are shown. See Supplementary Table 1 for specimen numbers registered in Osaka Museum of Natural History, Osaka (OSA-TB).

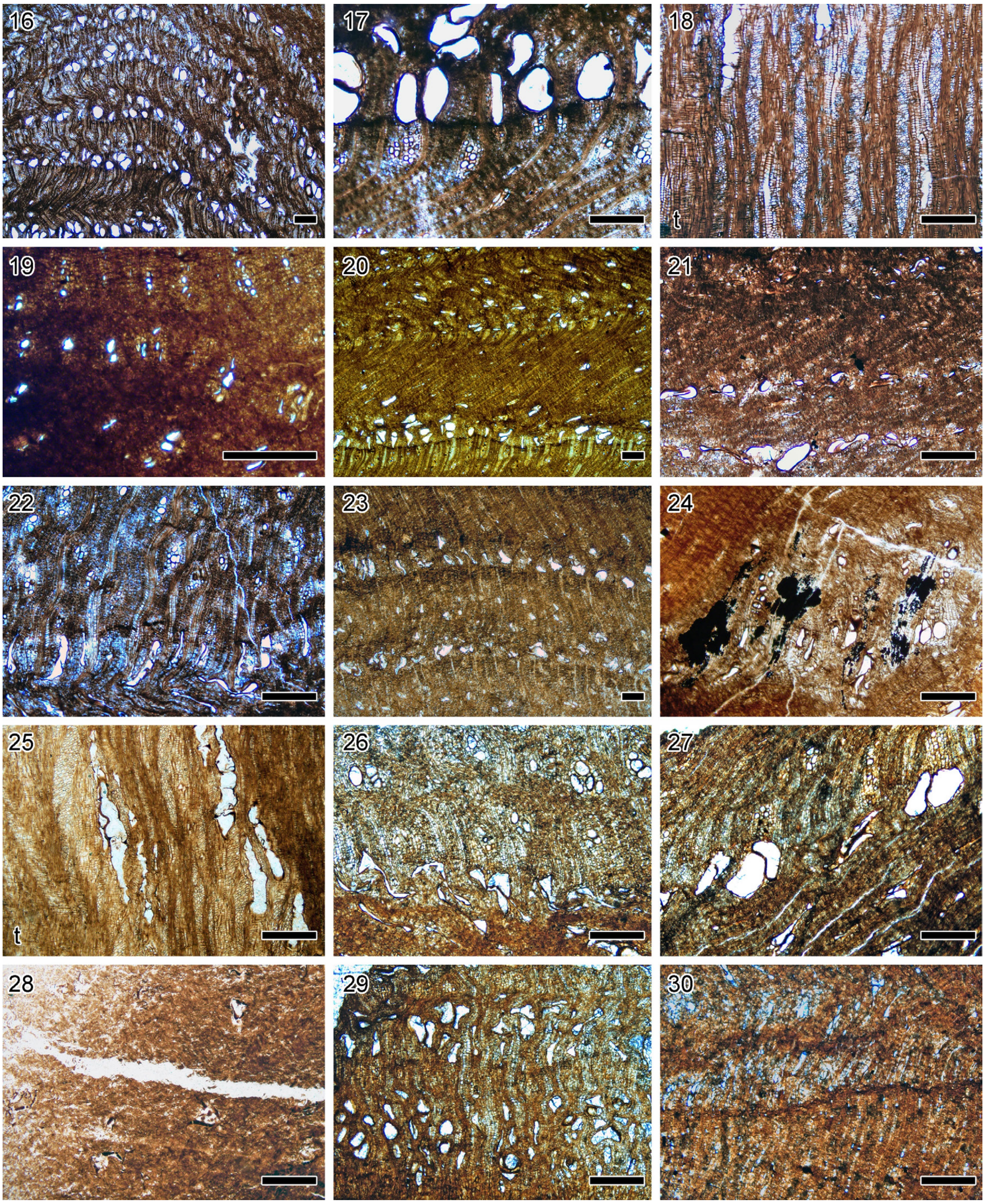

Supplementary Figure 4. *Wataria parvipora* from the Nakamura Formation in the PFP section. Numbers correspond to individual numbers in Supplementary Figure 1. Scale bars are 250 μm. Cross-sections are shown unless a panel is labeled with “t” (tangential-section). See Supplementary Table 1 for specimen numbers registered in Osaka Museum of Natural History, Osaka (OSA-TB).

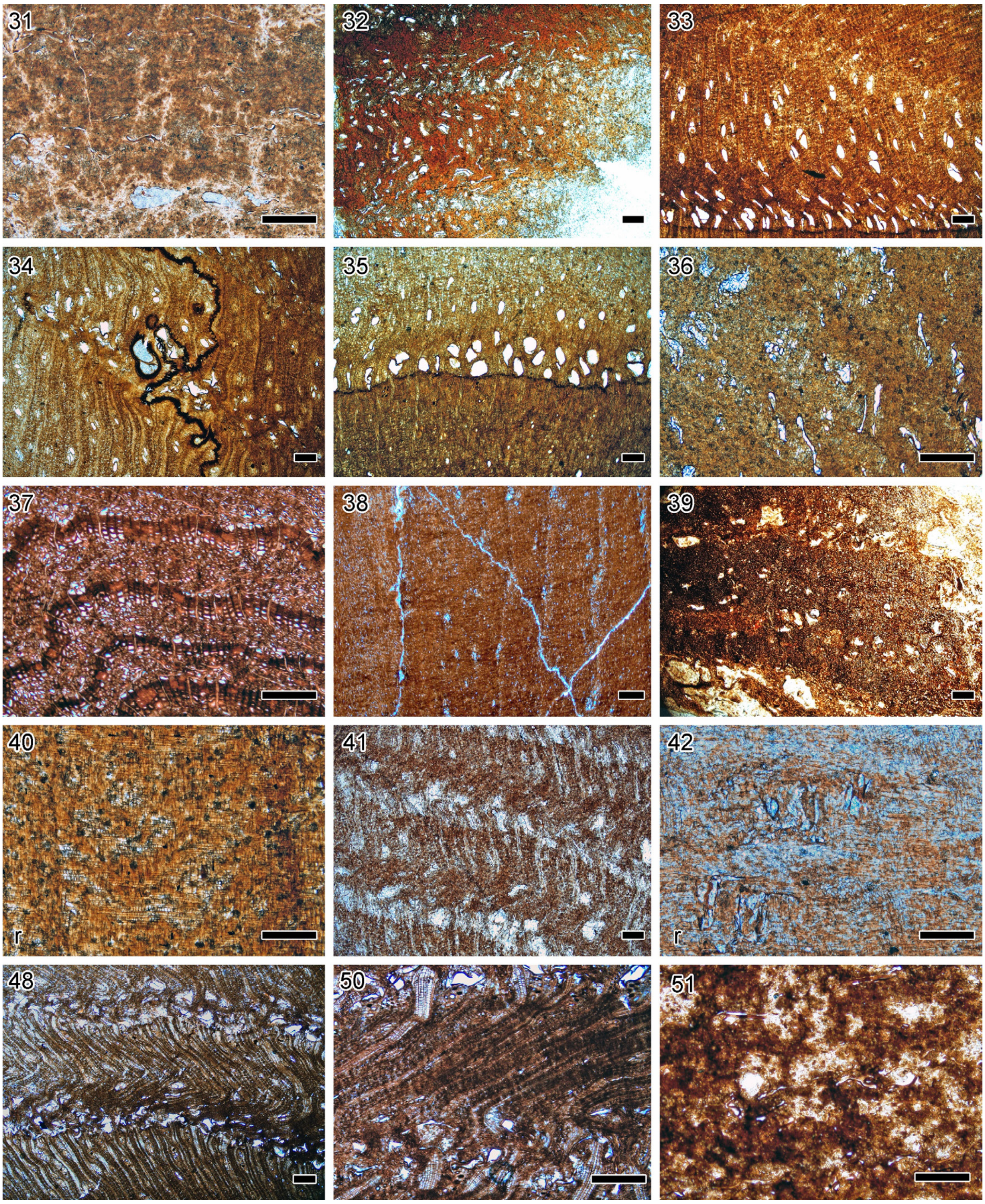

Supplementary Figure 5. *Wataria parvipora* from the Nakamura Formation in the PFP section. Numbers correspond to individual numbers in Supplementary Figure 1. Scale bars are 250 µm. Cross-sections are shown unless a panel is labeled with “r” (radial-section). See Supplementary Table 1 for specimen numbers registered in Osaka Museum of Natural History, Osaka (OSA-TB).

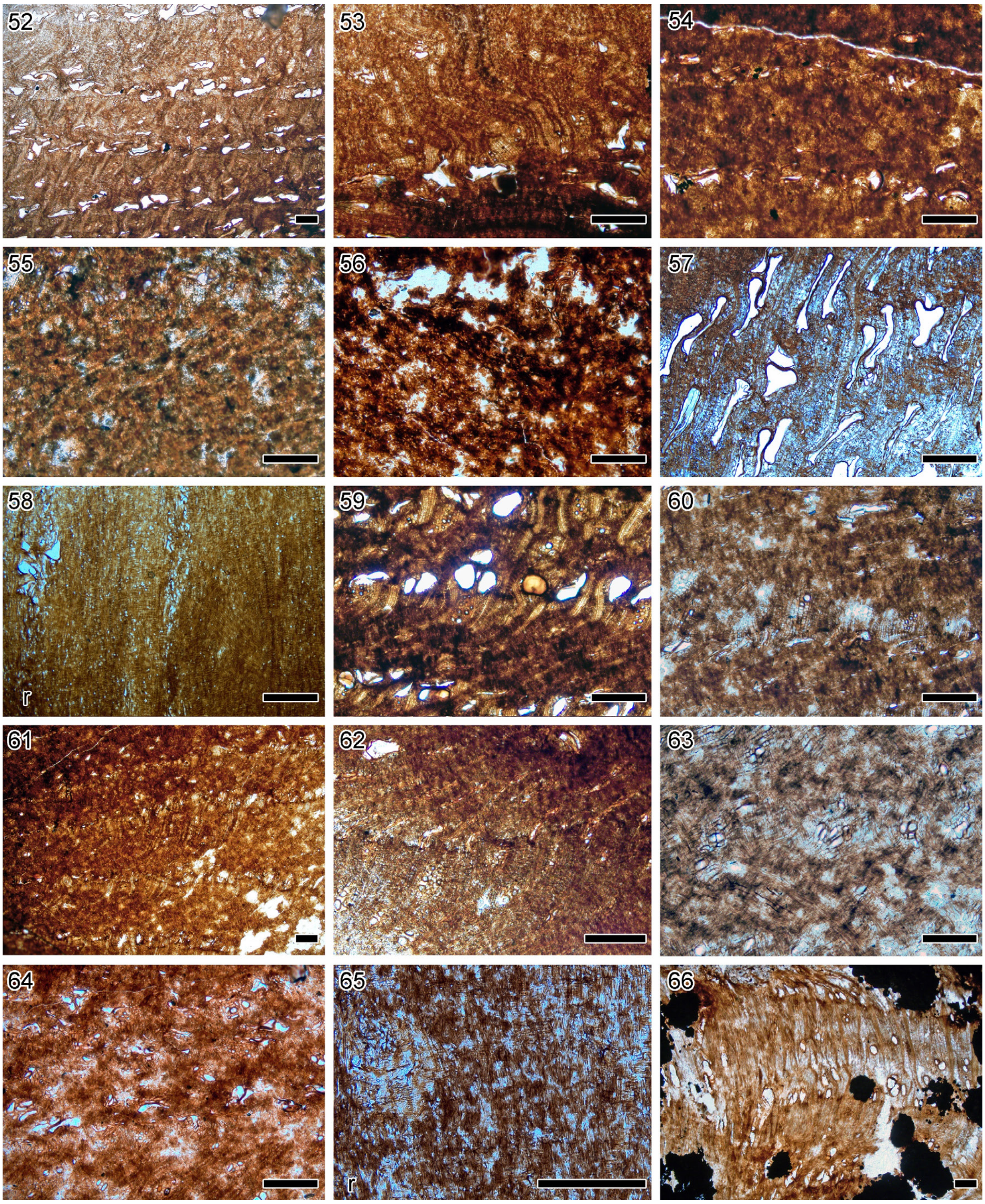

Supplementary Figure 6. *Wataria parvipora* from the Nakamura Formation in the PFP section. Numbers correspond to individual numbers in Supplementary Figure 1. Scale bars are 250 μm. Cross-sections are shown unless a panel is labeled with “r” (radial-section). See Supplementary Table 1 for specimen numbers registered in Osaka Museum of Natural History, Osaka (OSA-TB).

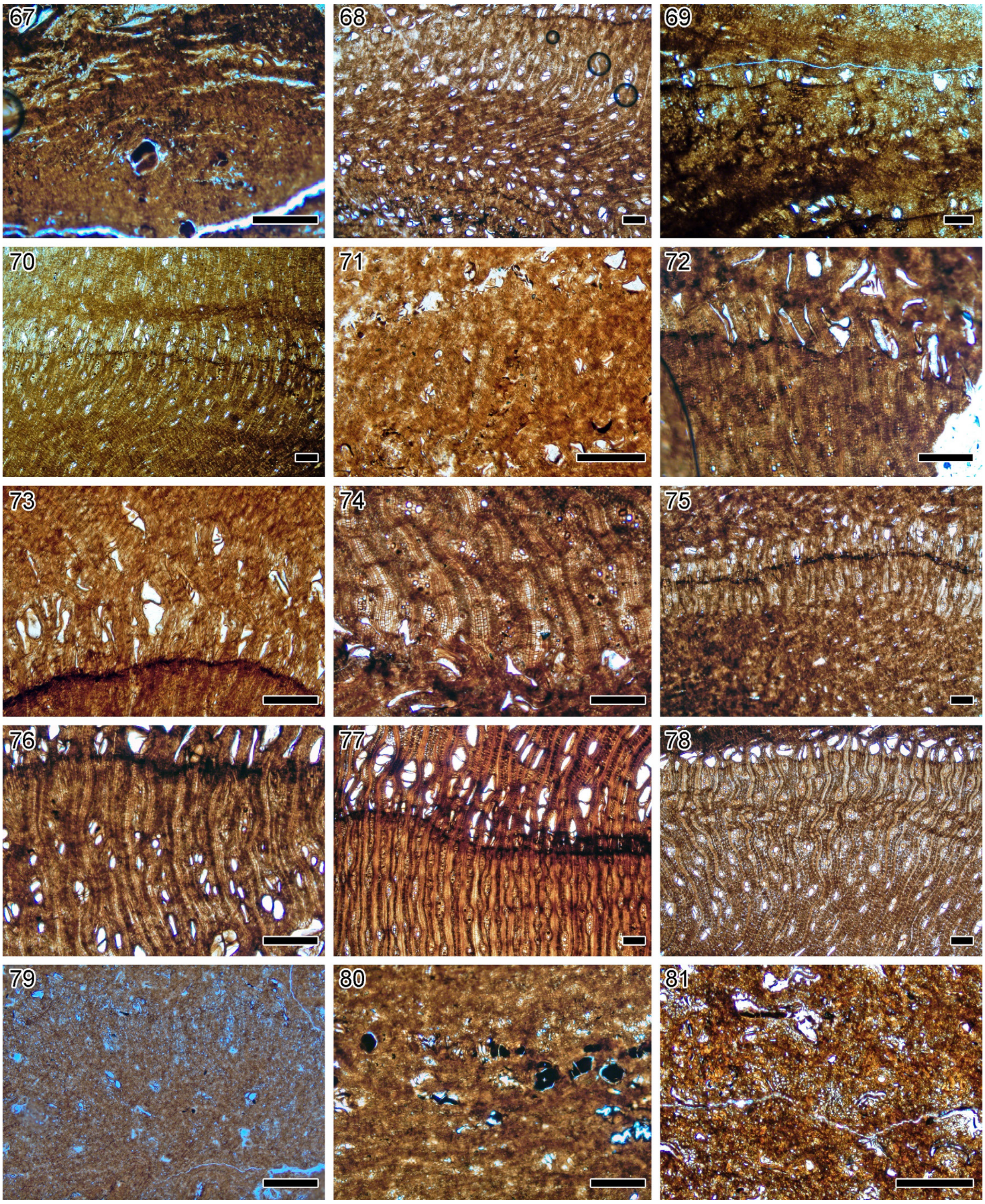

Supplementary Figure 7. *Wataria parvipora* from the Nakamura Formation in the PFP section. Numbers correspond to individual numbers in Supplementary Figure 1. Scale bars are 250 µm. Cross-sections are shown. See Supplementary Table 1 for specimen numbers registered in Osaka Museum of Natural History, Osaka (OSA-TB).

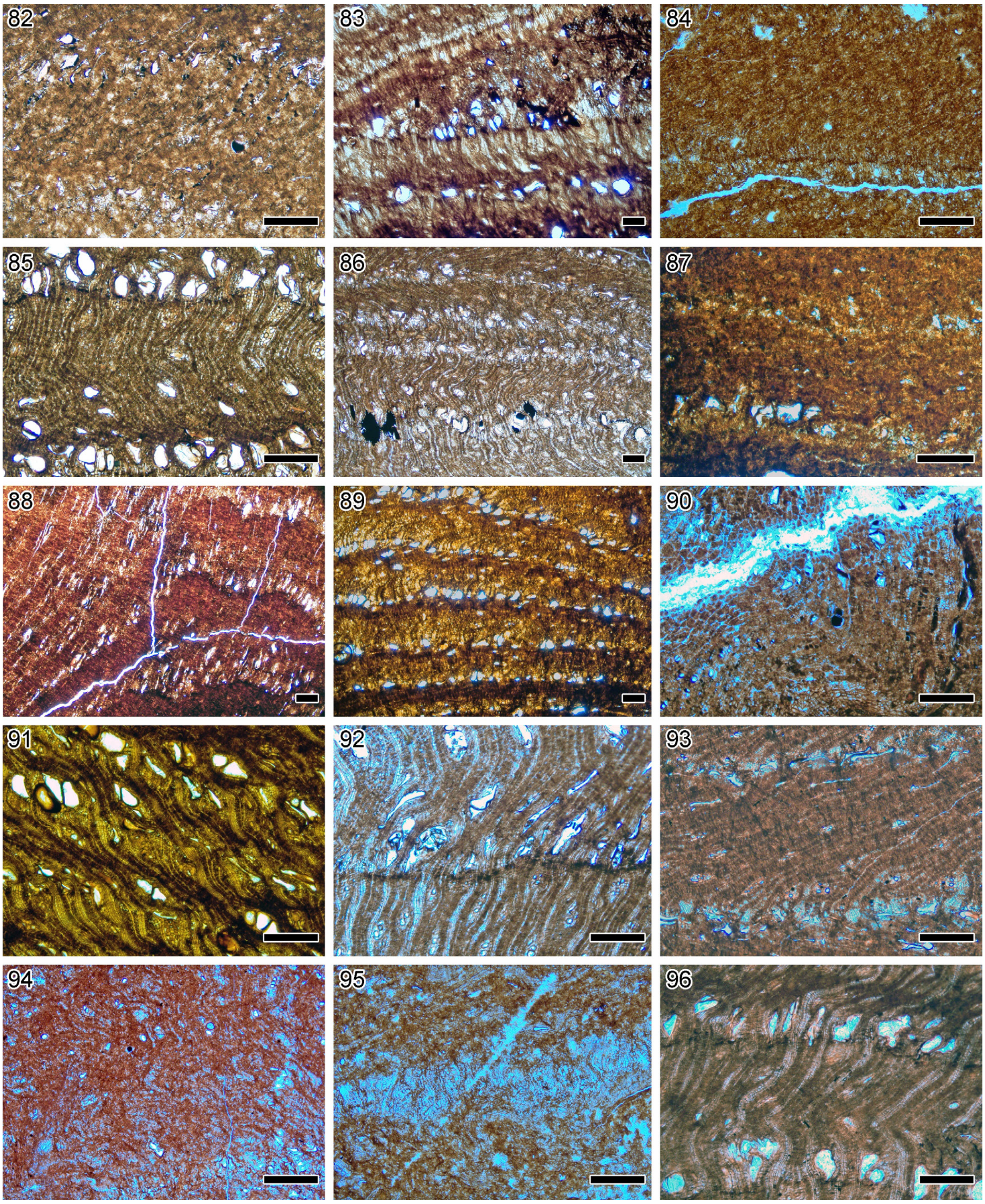

Supplementary Figure 8. *Wataria parvipora* from the Nakamura Formation in the PFP section. Numbers correspond to individual numbers in Supplementary Figure 1. Scale bars are 250 μm. Cross-sections are shown. See Supplementary Table 1 for specimen numbers registered in Osaka Museum of Natural History, Osaka (OSA-TB).

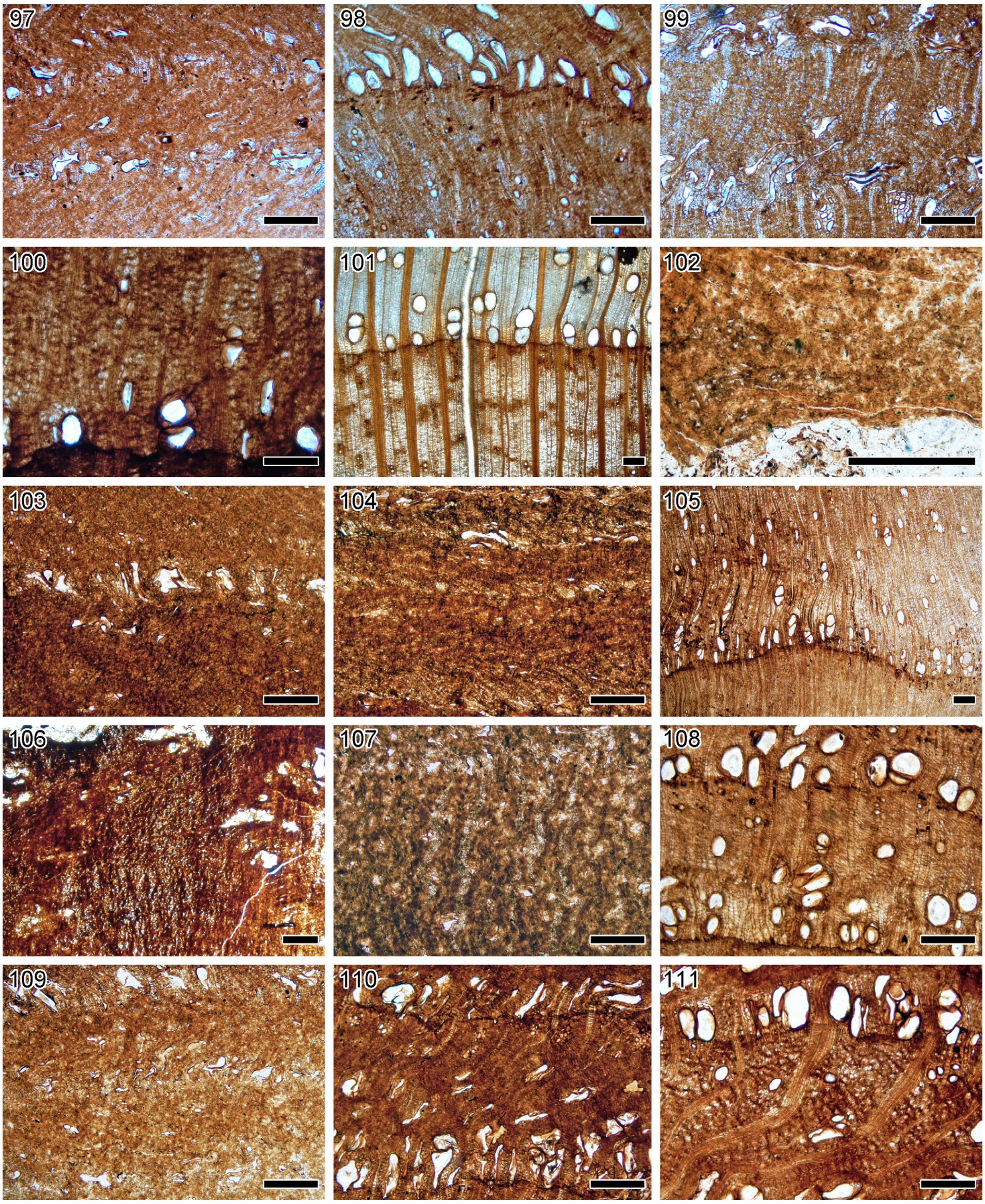

Supplementary Figure 9. *Wataria parvipora* from the Nakamura Formation in the PFP section. Numbers correspond to individual numbers in Supplementary Figure 1. Scale bars are 250 µm. Cross-sections are shown. See Supplementary Table 1 for specimen numbers registered in Osaka Museum of Natural History, Osaka (OSA-TB).

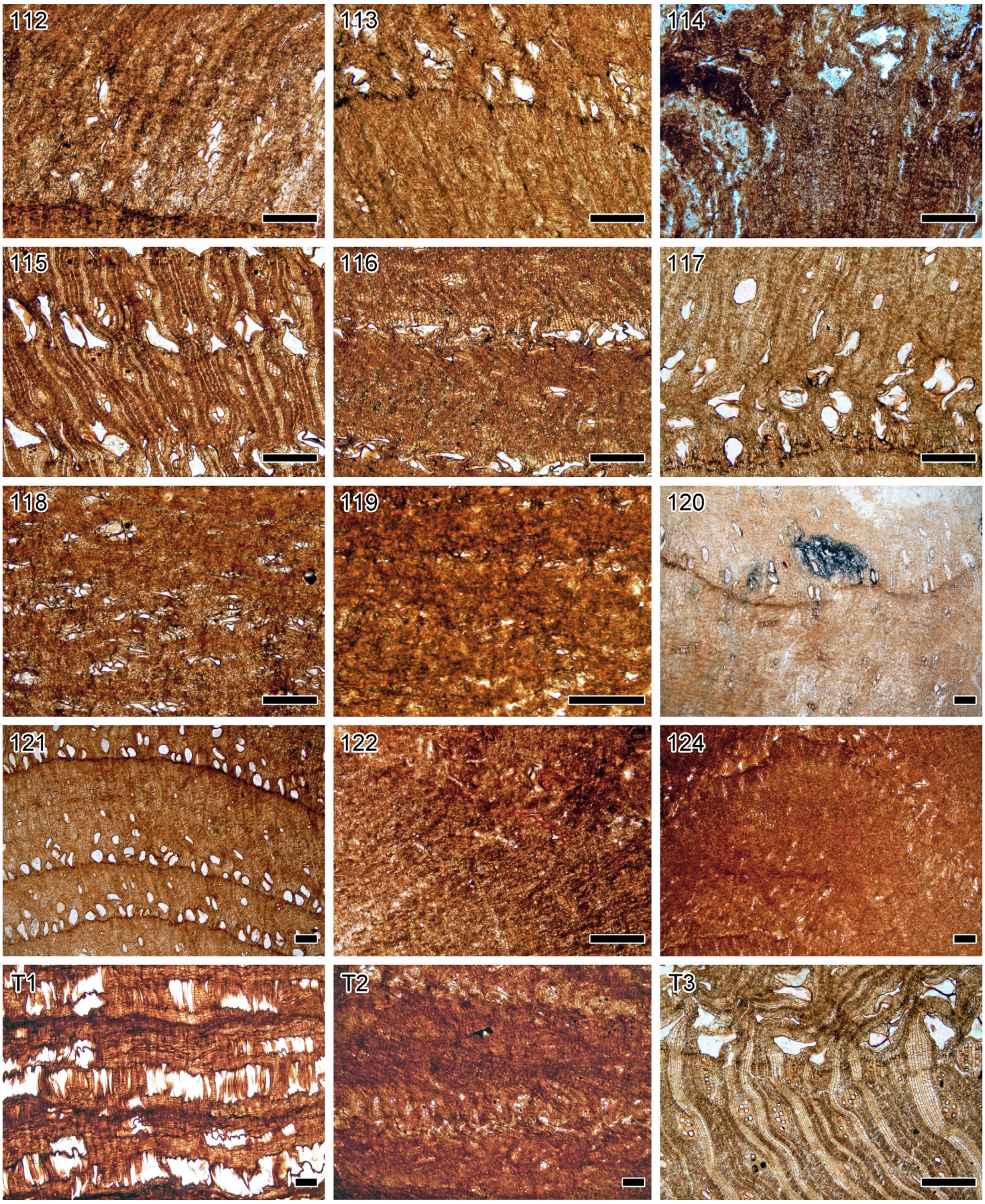

Supplementary Figure 10. *Wataria parvipora* from the Nakamura Formation in the PFP section. Numbers correspond to individual numbers in Supplementary Figure 1. Scale bars are 250 µm. Cross-sections are shown. See Supplementary Table 1 for specimen numbers registered in Osaka Museum of Natural History, Osaka (OSA-TB).

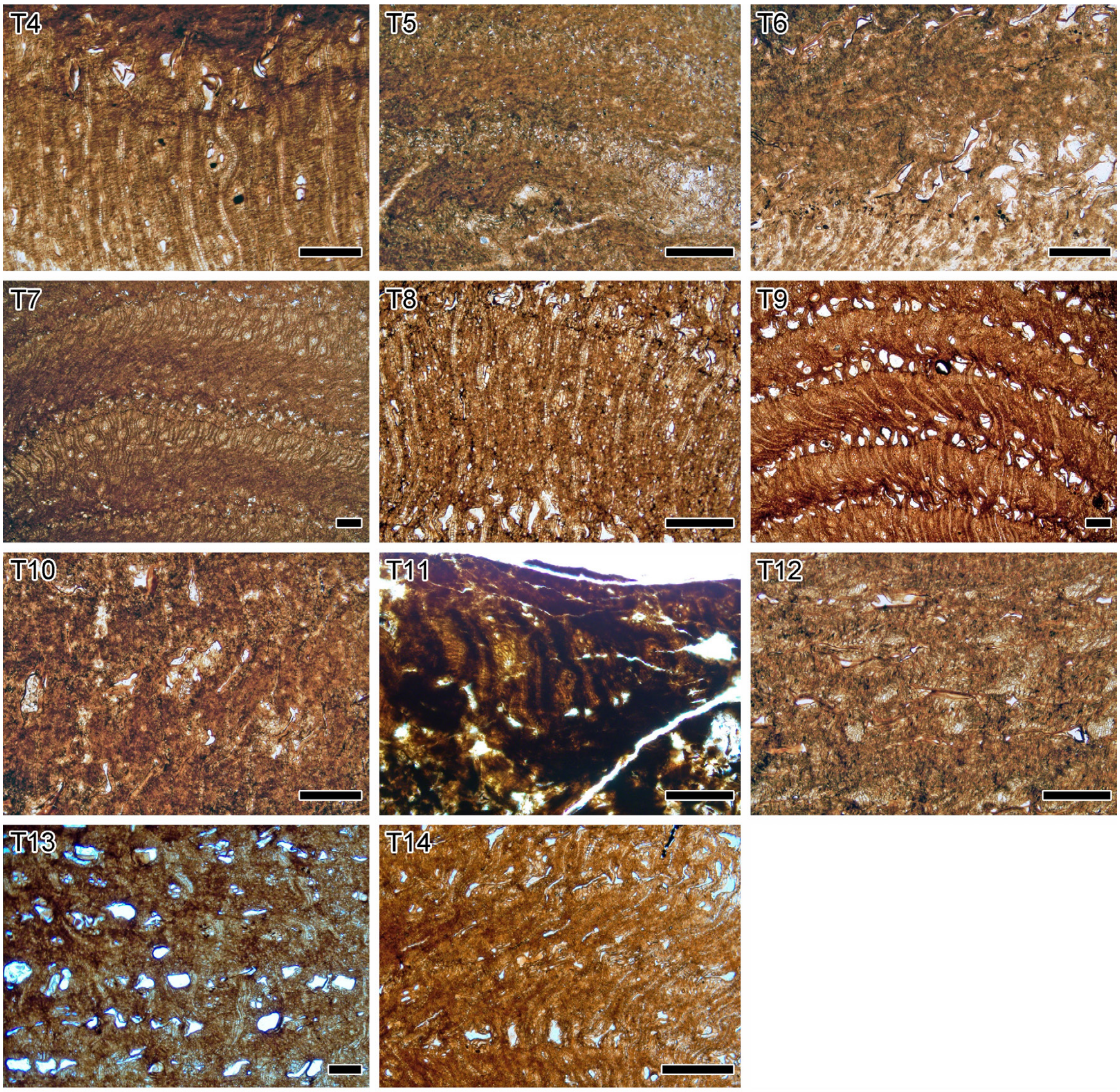

Supplementary Figure 11. *Wataria parvipora* from the Nakamura Formation in the PFP section. Numbers correspond to individual numbers in Supplementary Figure 1. Scale bars are 250 μm. Cross-sections are shown. See Supplementary Table 1 for specimen numbers registered in Osaka Museum of Natural History, Osaka (OSA-TB).

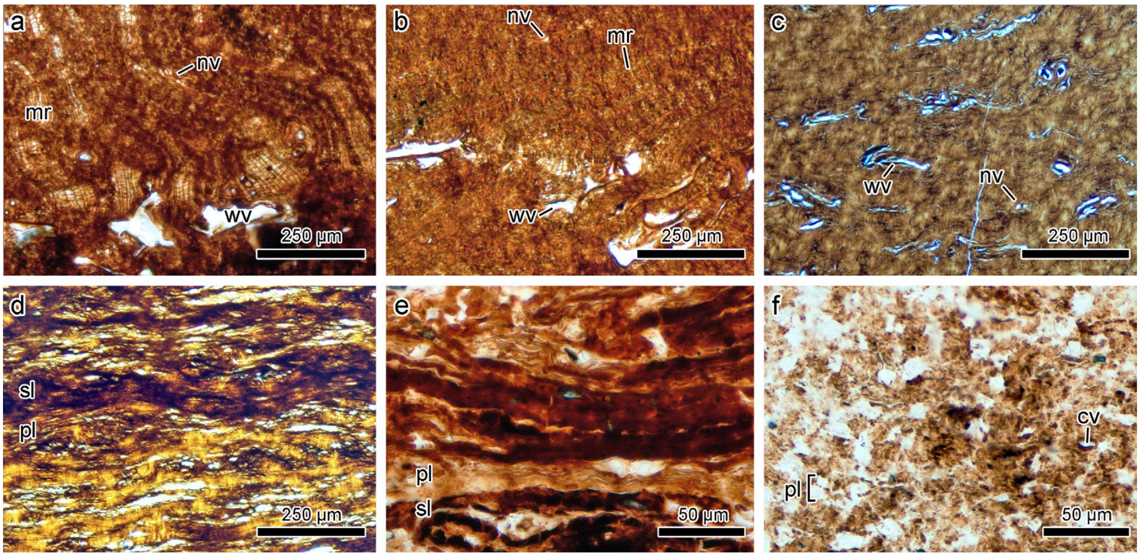

Supplementary Figure 12. Variations of preservation found in cross-sections of *Wataria* woods (**a**) Radial cell files are deformed, but most cells retain original shapes except for wide vessels. mr, maltiseriate ray; nv, narrow vessel; wv, wide vessel. Individual #53. OSA-TB 9156–c1. (**b**) Cells are deformed. Rays are brighter in color than other radial cell files, forming alternation of bright and dark parts. Individual #53. OSA-TB 9156–c1. (**c**) Small cells are crushed to form radial lineations. Wide vessels and clusters of narrow vessels are also compacted in a radial direction, but cavities of vessels are recognizable. Alternation of bright and dark parts are visible. Individual #2. OSA-TB 9105–c1. (**d**) Early and late woods are compacted to form porous (pl) and solid (sl) layers respectively. Radial lineations are visible. Individual #2. OSA-TB 9105–c1. (**e**) Porous and solid layers are visible, but cavities of vessels are almost lost by compaction. OSA-TB 9105–c3. (**f**) Crushed vessels (cv) are visible, but other tissues are degradated. Individual #2. OSA-TB 9105–c3.

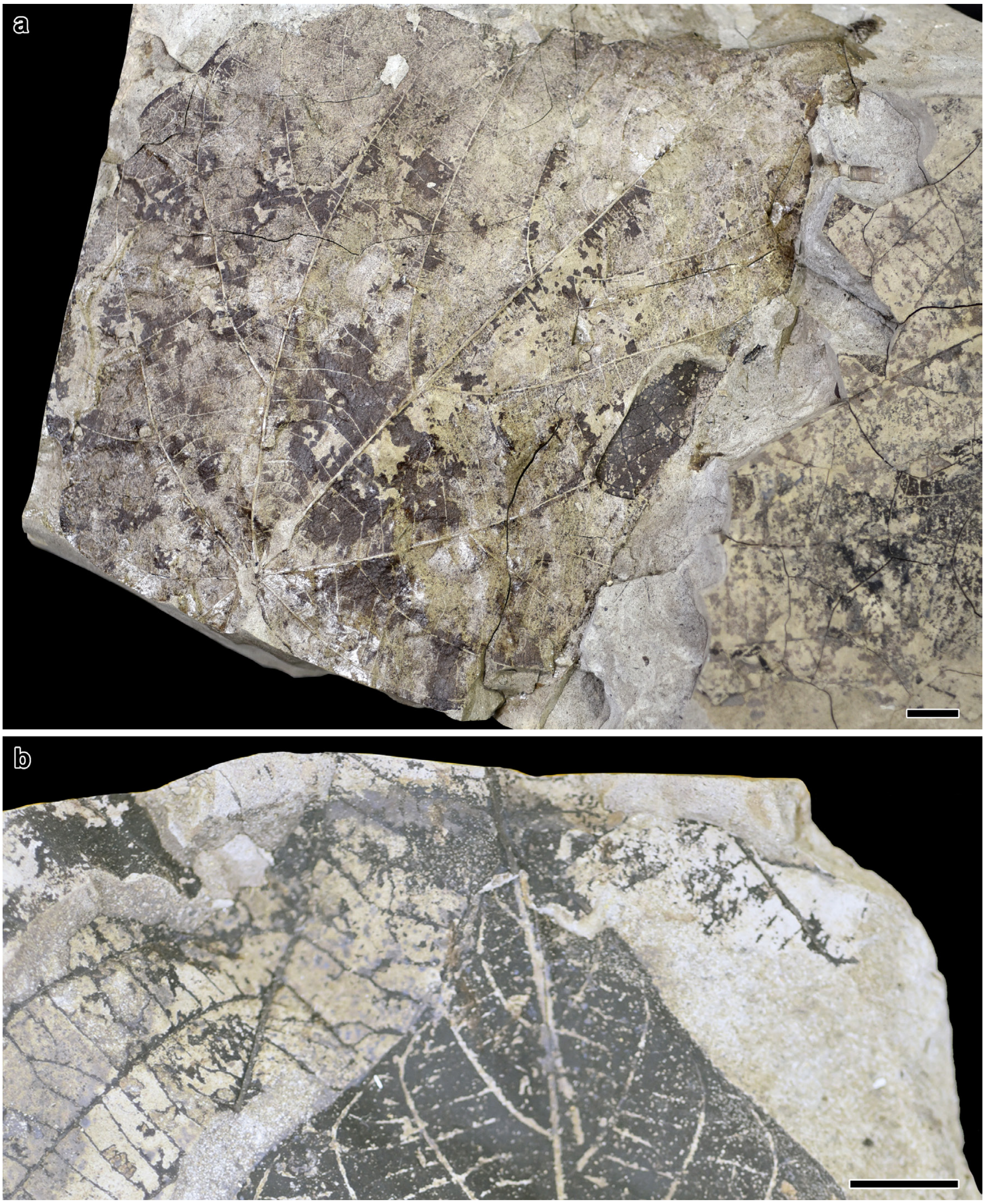

Supplementary Figure 13. *Byttneriophyllum tiliifolium* from the Nakamura Formation. (**a**) OSA TB 9243–2 from site 3. (**b**) OSA TB 9100 from loc. Otb001. Scale bars are 1 cm.

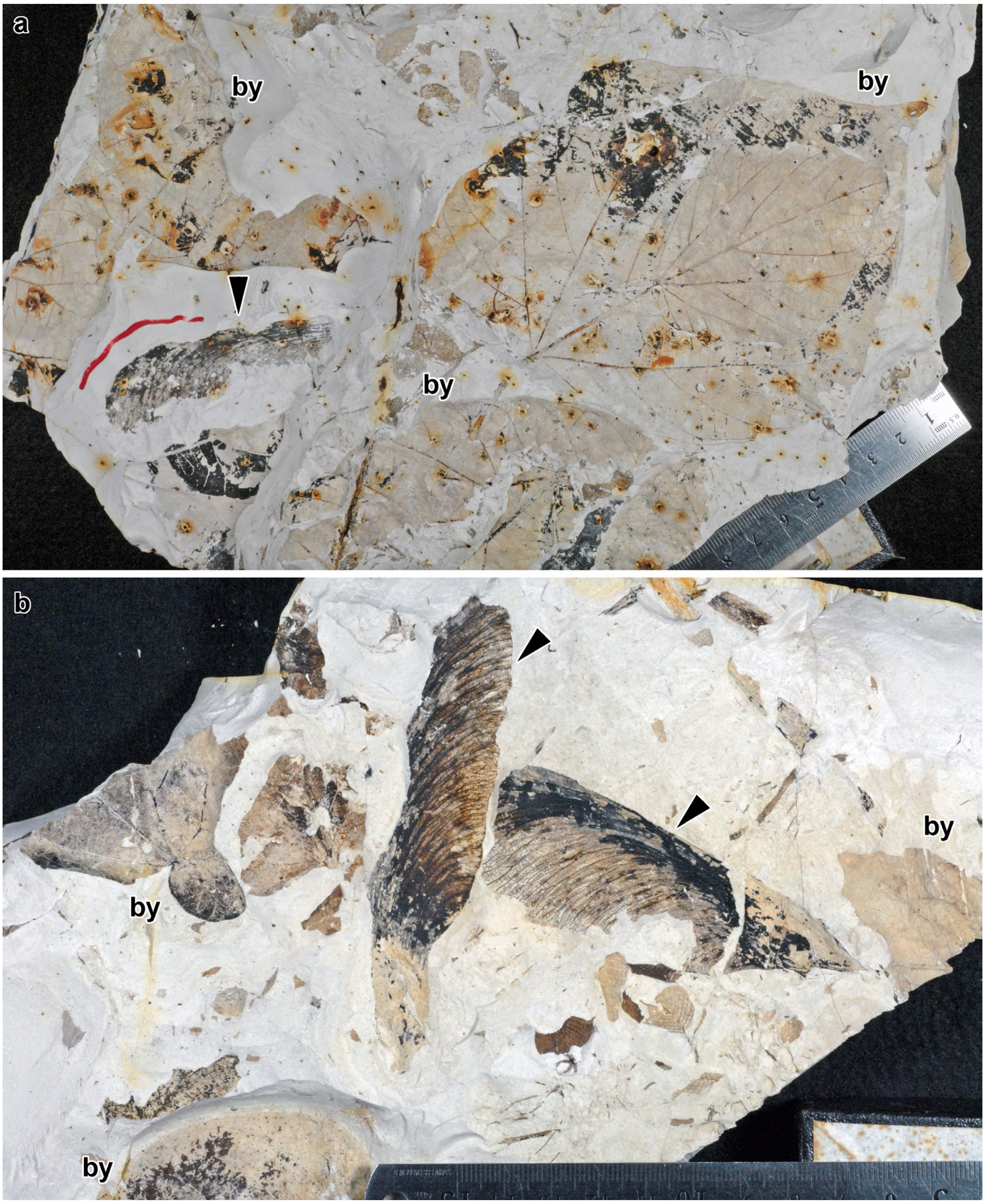

Supplementary Figure 14. *Banisteriaecarpum giganteum* from the Nakamura Formation distributed in Hirakaido, Kani, Gifu Prefecture, central Japan. (**a**) NSM-PP-23949. (**b**) NSM-PP-23947. Note that *Ba. giganteum* samaras (arrowheads) occur with *Byttneriophyllum tiliifolium* leaves (by).

Supplementary note

Family: MALVACEAE Juss., 1789[1]

Fossil-genus: **Byttneriophyllum** Givulescu ex Knobloch et Kvaček, 1965[2]

Fossil-species: *Byttneriophyllum tiliifolium* (A. Braun) Knobloch et Kvaček, 1965[2]

Specimens: OSA-TB 9243–1 (Fig. 6a, b), –2 (Supplementary Fig. 13a), –3 (Fig. 6c); OSA-TB 9100 (Supplementary Fig. 13b)

Description: The leaves are prominently asymmetrical. The lamina is entire-margined, unlobed with cordate base and acuminate apex, kidney-shape, 40–220 mm long and 30–180 mm wide (Fig. 6a, b, Supplementary Fig. 13a, b). The primary veins actinodromously separate into five to seven branches at the base. The longest primary vein obliquely traverses the lamina to the apex (Fig. 6a, b, Supplementary Fig. 13a). The basalmost pair of primary veins gives off secondary veins in an agrophic manner, while either or both of the second basal primary veins form secondary veins only in the exmedial direction. The secondary veins are brochidodromous, forming loops with adjacent primary or secondary veins (Fig. 6a, b, Supplementary Fig. 13a). The percurrent tertiary veins develop oppositely and alternately. The tertiary veins often run outwardly from secondary veins constituting a loop, which ends on the leaf margin (Fig. 6a, b, Supplementary Fig. 13a). The quaternary veins diverge perpendicularly from the tertiary veins (Fig. 6a, b, Supplementary Fig. 13a). The higher-order veins form areoles. Areoles usually do not encircle a veinlet, but one veinlet occasionally enters into the areole (Fig. 6c).

Remarks: Although epidermal characters are not available for our leaf specimens, the leaf shape and venation patterns suggest that they are *B. tiliifolium*[3]. Leaves of this type were classified as “*Alangium aequalifolium*” in the Mizunami Group[4, 5], but this fossil-species is a synonym of *B. tiliifolium*[2,3]. Leaves of the extant *Alangium* usually have profusely branching veinlets in the areoles[6] in contrast to simple veinlets in *B.*

tiliifolium[3].

Fossil-genus: **Wataria** K. Terada et M. Suzuki, 1998[6]

Fossil-species: *Wataria parvipora* K. Terada et M. Suzuki, 1998[6]

Specimens: OSA-TB 9204–c1 (Fig. 5a, Supplementary Figure 2a, b), –c2 (Supplementary Figure 2e), –r1 (Fig. 5b, c, Supplementary Figure 2c, d), –t1 (Fig. 5d). See Supplementary Table 1 for other specimens examined.

Description: Wood is ring porous with distinct growth rings (Fig. 5a, Supplementary Figure 2a, b). Larger vessels are arranged in zones of one or two layers in earlywood. They are mostly solitary, but some occur in radial or oblique multiples of two or three, and 58–188 (mean 131) μm in vertical length. Solitary vessels are round or somewhat radially elongated oval in cross-section, and 86–253 (mean 143) μm in their maximum diameter (Fig. 5a, Supplementary Figure 2a–c). Groups of up to 10 narrow vessels are surrounded by one- to three-seriate vasicentric parenchyma in late wood (Supplementary Figure 2a, b). They are circular in outline, 13–82 (mean 35) μm in maximum diameter, very thick-walled, around 3 μm thick and 64–278 (mean 140) μm in vertical length (Supplementary Figure 2a, b, d). The perforation plates are exclusively simple (Supplementary Figure 2c, d). The intervessel pits are alternate and dense. The helical thickenings are absent (Supplementary Figure 2c, d). The fibers are square or polygonal in cross-section and mostly constitute the ground mass of the latewood. The wood parenchyma are apotracheal in latewood and mostly constitute the ground mass of the earlywood. Apotracheal parenchyma form uni- or bi-seriate tangential bands that alternate with one- to three-seriate rows of fibers (Supplementary Figure 2a, b). The axial parenchyma are conspicuously storied (Fig. 5b) in strands with two to eight cells. The rays are one to seven seriate (Fig. 5b). The multiseriate rays are mostly five to seven cells wide and consist of procumbent cells and tile cells (Fig. 5d). The procumbent cells are 6.1–21 (mean 12) μm, 5.6–13 (mean 8.5) μm, and 17–70 (mean

40) µm in the tangential, vertical, and radial diameters, respectively (Fig. 5c). The tile cells are 7.9–25 (mean 13) µm, 5.1–25 (mean 12) µm, and 5.1–19 (mean 10) µm in tangential, vertical, and radial diameters, respectively (Fig. 5c). Of the 100 cells examined, 99 vertical diameters are < 12 µm in the procumbent cells, and 57 vertical diameters are < 12 µm in the tile cells. Thin-walled tyloses often develop in wider vessels. Gum-like deposits are found in some narrow vessels and crystals are absent (Supplementary Figure 2e).

Remarks: The obtained wood fragments are somewhat deformed due to the close position to the roots. About the half of the tile cells have vertical diameters close to those of the procumbent cells, while the remaining half have longer ones. Therefore, the tile cells of our specimens are classified as *Pterospermum*-type and intermediate of the *Durio*- and *Pterospermum*-types[7]. In addition, we could observe multistoried axial parenchyma and uni- or bi-seriate tangential bands of apotracheal parenchyma alternating with one- to three-seriate fiber rows. These characters suggest that our specimens could be *Wataria*[6]. We assigned them to *W. parvipora* based on their narrower early wood vessels than are found in *W. miocenica* and narrower pore zone than in *W. oligocenica*[6].

Fossil-genus: **Banisteriaecarpum** (Göppert) Kräusel, 1951[8]

Fossil-species: *Banisteriaecarpum giganteum* (Göppert) Kräusel, 1951[8]

Specimens: NSM-PP-23949 (Supplementary Figure 14a), -23947 (Supplementary Figure 14b).

Localities: Hirakaido, Kani City, Gifu Pref., Japan (35°26′10″ N, 137°5′21″ E), where the upper part of the Nakamura Formation is outcropped[9].

Description: Samaras are ribbon-shape, up to 100 mm long and 30 mm wide (Supplementary Figure 14a, b). The wing is adnate to a distal side of ovary, papery, narrowing at the basal one-third (Supplementary Figure 14b). The ovary is oblong-

obovate, ca. 10 mm long and ca. 20 mm wide (Supplementary Figure 14b). The primary veins radiate from the wing base and run parallelly along the dorsal margin. They curve to traverse the wing and branch several times before reaching to the ventral margin (Supplementary Figure 14a, b). The secondary veins diverge perpendicularly from the primary veins, connecting adjacent primary veins to form a mesh (Supplementary Figure 14b).

Remarks: Our samaras have a large wing, with primary veins radiating from the wing base, and a short peduncle, which characterizes *Banisteriaecarpum giganteum*[8,10].

References

1. Jussieu, A. L. de. Malvaceae. In *Genera Plantarum* 271–279 (1789).
2. Knobloch, E. & Kvaček, Z. *Byttneriophyllum tiliaefolium* (Al. Braun) Knobloch et Kvaček in den tertiären Floren der Nordhalbkugel. *Sb. Geol. Ved. Paleontol.* **5**, 123–166 (1965).
3. Worobiec, G., Worobiec, E. & Kvaček, Z. Neogene leaf morphotaxa of Malvaceae s.l. in Europe. *Int. J. Plant Sci.* **171**, 892–914 (2010).
4. Tokunaga, S. & Onoe, T. Report of the paleobotanical study on the main coal seams in the Toki and Kani districts of the Mino Lignite Field, Gifu Prefecture and in the Miike and Amakusa Coal Fields, Kyushu. *Bull. Geol. Surv. Japan* **11**, 577–584 (1960).
5. Tanai, T. The revision of the so-called *Alangium* leaves from the Paleogene of Hokkaido, Japan. *Bull. Natl. Sci. Mus. Tokyo Ser. C* **15**, 121–149 (1989).
6. Terada, K. & Suzuki, M. Revision of the so-called "*Reevesia*" fossil woods from the Tertiary in Japan– A proposal of new genus *Wataria* (Sterculiaceae). *Rev. Palaeobot. Palynol.* **98**, 207–222 (1998).
7. Chattaway, M.M. Tile-cells in the rays of the Malvales. *New Phytol.* **32**, 261–353 (1933).
8. Kräusel, R. Die tertiäre "Riesenahorn" *Banisteriaecarpum* nov. gen. Abh. Senckenb. Naturforsch. Ges. **485**, 75–80 (1951).
9. Shikano, K., 1995. Stratigraphy of the Nakamura Formation. In *Strata and fossils of Nakamura Formation in Minokamo Basin — Reports for fossil footprints of*

mammals and fossil forests (ed. Minokamo City Educational Boards) 2–18 (Minokamo City Board of Education, 1995).

10. Kvaček, Z. & Hably, L. The whole plant reconstruction of *Banisteriaecarpum giganteum* and *Byttneriophyllum tiliifolium* - a preliminary report. *Folia Mus. rerum nat. Bohem. occident., Geol. Paleobiol.* **48**, 1–10 (2014).

Supplementary Table 1 Individual numbers and specimen numbers of *in situ* stumps studied.

Individual number	Species	Trunk diameter (cm)	Specimen number (wood)	Specimen number (slide)	Remarks
1	*Wataria* sp.	137	TB9104	–c1	Supplementary Fig. 3
2	*Wataria* sp.	70	TB9105	–c1	Supplementary Figure 3, 12c, d
				–c3	Supplementary Figure 12e, f
3	*Wataria* sp.	72	TB9106	–c1	Supplementary Fig.3
4	*Wataria parvipora*	81	TB9107	–c1	Supplementary Fig.3
5	*Wataria parvipora*	36	TB9108	–c1	Supplementary Fig.3
6	*Wataria parvipora*	38	TB9109	–c1	Supplementary Fig.3
7	*Wataria parvipora*	32	TB9110	–c1	Supplementary Fig.3
8	*Wataria parvipora*	89	TB9111	–c1	Supplementary Fig.3
9	*Wataria parvipora*	28	TB9112	–c1	Supplementary Fig.3
10	*Wataria parvipora*	66	TB9113	–c1	Supplementary Fig.3
11	*Wataria parvipora*	26	TB9114	–c1	Supplementary Fig.3
12	*Wataria parvipora*	43	TB9115	–c1	Supplementary Fig.3
13	*Wataria parvipora*	19	TB9116	–c1	Supplementary Fig.3
14	*Wataria parvipora*	100	TB9117	–c1	Supplementary Fig.3
15	*Wataria parvipora*	20	TB9118	–c1	Supplementary Fig.3
16	*Wataria parvipora*	43	TB9119	–c1	Supplementary Fig. 4
17	*Wataria parvipora*	39	TB9120	–c1	Supplementary Fig. 4
18	*Wataria parvipora*	45	TB9121	–t1	Supplementary Fig. 4
19	*Wataria parvipora*	72	TB9122	–c1	Supplementary Fig. 4
20	*Wataria parvipora*	29	TB9123	–c1	Supplementary Fig. 4
21	*Wataria parvipora*	19	TB9124	–c1	Supplementary Fig. 4
22	*Wataria parvipora*	23	TB9125	–c1	Supplementary Fig. 4
23	*Wataria parvipora*	28	TB9126	–c1	Supplementary Fig. 4
24	*Wataria parvipora*	39	TB9127	–c1	Supplementary Fig. 4
25	*Wataria parvipora*	21	TB9128	–t1	Supplementary Fig. 4
26	*Wataria parvipora*	33	TB9129	–c1	Supplementary Fig. 4
27	*Wataria parvipora*	37	TB9130	–c1	Supplementary Fig. 4
28	*Wataria* sp.	15	TB9131	–c1	Supplementary Fig. 4
29	*Wataria parvipora*	13	TB9132	–c1	Supplementary Fig. 4
30	*Wataria parvipora*	23	TB9133	–c1	Supplementary Fig. 4
31	*Wataria* sp.	33	TB9134	–c1	Supplementary Fig. 5
32	*Wataria parvipora*	26	TB9135	–c1	Supplementary Fig. 5
33	*Wataria parvipora*	30	TB9136	–c1	Supplementary Fig. 5
34	*Wataria parvipora*	52	TB9137	–c1	Supplementary Fig. 5
35	*Wataria parvipora*	31	TB9138	–c1	Supplementary Fig. 5
36	*Wataria parvipora*	61	TB9139	–c1	Supplementary Fig. 5
37	*Taxodioxylon* sp.	67	TB9140	–c1	Supplementary Fig. 5
38	*Wataria parvipora*	30	TB9141	–c1	Supplementary Fig. 5
39	*Wataria parvipora*	25	TB9142	–c1	Supplementary Fig. 5
40	*Wataria parvipora*	40	TB9143	–c1	Supplementary Fig. 5
41	*Wataria parvipora*	56	TB9144	–c1	Supplementary Fig. 5
42	*Wataria parvipora*	53	TB9145	–r1	Supplementary Fig. 5
48	*Wataria parvipora*	30	TB9151	–c1	Supplementary Fig. 5
50	*Wataria parvipora*	30	TB9153	–c1	Supplementary Fig. 5
51	*Wataria* sp.	34	TB9154	–c1	Supplementary Fig. 5
52	*Wataria parvipora*	54	TB9155	–c1	Supplementary Fig. 6
53	*Wataria parvipora*	15	TB9156	–c1	Supplementary Fig. 6, 12a, b

Supplementary Table 1 (Continued)

Individual number	Species	Trunk diameter (cm)	Specimen number (wood)	Specimen number (slide)	Remarks
54	*Wataria parvipora*	11	TB9157	–c1	Supplementary Fig. 6
55	*Wataria parvipora*	12	TB9158	–c1	Supplementary Fig. 6
56	*Wataria parvipora*	16	TB9159	–c1	Supplementary Fig. 6
57	*Wataria parvipora*	27	TB9160	–c1	Supplementary Fig. 6
58	*Wataria parvipora*	11	TB9161	–c1	Supplementary Fig. 6
59	*Wataria parvipora*	120	TB9162	–c1	Supplementary Fig. 6
60	*Wataria parvipora*	10.5	TB9163	–c1	Supplementary Fig. 6
61	*Wataria parvipora*	6	TB9164	–c1	Supplementary Fig. 6
62	*Wataria parvipora*	24	TB9165	–c1	Supplementary Fig. 6
63	*Wataria parvipora*	21	TB9166	–c1	Supplementary Fig. 6
64	*Wataria parvipora*	28	TB9167	–c1	Supplementary Fig. 6
65	*Wataria* sp.	26	TB9168	–r1	Supplementary Fig. 6
66	*Wataria parvipora*	18	TB9169	–c1	Supplementary Fig. 6
67	*Wataria* sp.	16	TB9170	–c1	Supplementary Fig. 7
68	*Wataria parvipora*	10	TB9171	–c1	Supplementary Fig. 7
69	*Wataria parvipora*	13	TB9172	–c1	Supplementary Fig. 7
70	*Wataria parvipora*	28	TB9173	–c1	Supplementary Fig. 7
71	*Wataria parvipora*	7	TB9174	–c1	Supplementary Fig. 7
72	*Wataria parvipora*	13	TB9175	–c1	Supplementary Fig. 7
73	*Wataria parvipora*	9	TB9176	–c1	Supplementary Fig. 7
74	*Wataria parvipora*	30	TB9177	–c1	Supplementary Fig. 7
75	*Wataria parvipora*	8	TB9178	–c1	Supplementary Fig. 7
76	*Wataria parvipora*	20	TB9179	–c1	Supplementary Fig. 7
77	*Wataria parvipora*	39	TB9180	–c1	Supplementary Fig. 7
78	*Wataria parvipora*	11	TB9181	–c1	Supplementary Fig. 7
79	*Wataria parvipora*	6	TB9182	–c1	Supplementary Fig. 7
80	*Wataria parvipora*	7	TB9183	–c1	Supplementary Fig. 7
81	*Wataria parvipora*	7	TB9184	–c1	Supplementary Fig. 7
82	*Wataria parvipora*	7	TB9185	–c1	Supplementary Fig. 8
83	*Wataria parvipora*	6	TB9186	–c1	Supplementary Fig. 8
84	*Wataria parvipora*	6	TB9187	–c1	Supplementary Fig. 8
85	*Wataria parvipora*	4	TB9188	–c1	Supplementary Fig. 8
86	*Wataria parvipora*	4	TB9189	–c1	Supplementary Fig. 8
87	*Wataria parvipora*	12	TB9190	–c1	Supplementary Fig. 8
88	*Wataria parvipora*	13	TB9191	–c1	Supplementary Fig. 8
89	*Wataria parvipora*	11	TB9192	–c1	Supplementary Fig. 8
90	*Wataria* sp.	20	TB9193	–c1	Supplementary Fig. 8
91	*Wataria parvipora*	7	TB9194	–c1	Supplementary Fig. 8
92	*Wataria parvipora*	13	TB9195	–c1	Supplementary Fig. 8
93	*Wataria parvipora*	13	TB9196	–c1	Supplementary Fig. 8
94	*Wataria parvipora*	12	TB9197	–c1	Supplementary Fig. 8
95	*Wataria parvipora*	1	TB9198	–c1	Supplementary Fig. 8
96	*Wataria parvipora*	5	TB9199	–c1	Supplementary Fig. 8
97	*Wataria parvipora*	9	TB9200	–c1	Supplementary Fig. 9
98	*Wataria parvipora*	24	TB9201	–c1	Supplementary Fig. 9
99	*Wataria parvipora*	14	TB9202	–c1	Supplementary Fig. 9
100	*Wataria parvipora*	39	TB9203	–c1	Supplementary Fig. 9

Supplementary Table 1 (Continued)

Individual number	Species	Trunk diameter (cm)	Specimen number (wood)	Specimen number (slide)	Remarks
101	*Wataria parvipora*	16	TB9204	–c1	Fig. 5a, Supplementary Figs. 2a, b, 9
				–c2	Supplementary Fig. 2e
				–r1	Fig. 5b, d, Supplementary Fig. 2c, d
				–t1	Fig. 5c
102	*Wataria* sp.	15	TB9205	–c1	Supplementary Fig. 9
103	*Wataria parvipora*	6	TB9206	–c1	Supplementary Fig. 9
104	*Wataria parvipora*	10.5	TB9207	–c1	Supplementary Fig. 9
105	*Wataria parvipora*	10.5	TB9208	–c1	Supplementary Fig. 9
106	*Wataria* sp.	20	TB9209	–c1	Supplementary Fig. 9
107	*Wataria parvipora*	18	TB9210	–c1	Supplementary Fig. 9
108	*Wataria parvipora*	11	TB9211	–c1	Supplementary Fig. 9
109	*Wataria parvipora*	12	TB9212	–c1	Supplementary Fig. 9
110	*Wataria parvipora*	15	TB9213	–c1	Supplementary Fig. 9
111	*Wataria parvipora*	24	TB9214	–c1	Supplementary Fig. 9
112	*Wataria parvipora*	27	TB9215	–c1	Supplementary Fig. 10
113	*Wataria parvipora*	24	TB9216	–c1	Supplementary Fig. 10
114	*Wataria parvipora*	23	TB9217	–c1	Supplementary Fig. 10
115	*Wataria parvipora*	16	TB9218	–c1	Supplementary Fig. 10
116	*Wataria parvipora*	13	TB9219	–c1	Supplementary Fig. 10
117	*Wataria parvipora*	14	TB9220	–c1	Supplementary Fig. 10
118	*Wataria parvipora*	9	TB9221	–c1	Supplementary Fig. 10
119	*Wataria* sp.	11	TB9222	–c1	Supplementary Fig. 10
120	*Wataria parvipora*	14	TB9223	–c1	Supplementary Fig. 10
121	*Wataria parvipora*	11	TB9224	–c1	Supplementary Fig. 10
122	*Wataria* sp.	27	TB9225	–c1	Supplementary Fig. 10
124	*Wataria parvipora*	19	TB9227	–c1	Supplementary Fig. 10
T1	*Wataria parvipora*	42	TB9228	–c1	Supplementary Fig. 10
T2	*Wataria parvipora*	21	TB9229	–c1	Supplementary Fig. 10
T3	*Wataria parvipora*	20	TB9230	–c1	Supplementary Fig. 10
T4	*Wataria parvipora*	23	TB9231	–c1	Supplementary Fig. 11
T5	*Wataria* sp.	13	TB9232	–c1	Supplementary Fig. 11
T6	*Wataria parvipora*	15	TB9233	–c1	Supplementary Fig. 11
T7	*Wataria parvipora*	17	TB9234	–c1	Supplementary Fig. 11
T8	*Wataria parvipora*	3.5	TB9236	–c1	Supplementary Fig. 11
T9	*Wataria parvipora*	3	TB9237	–c1	Supplementary Fig. 11
T10	*Wataria parvipora*	14	TB9238	–c1	Supplementary Fig. 11
T11	*Wataria* sp.	10	TB9239	–c1	Supplementary Fig. 11
T12	*Wataria parvipora*	45	TB9240	–c1	Supplementary Fig. 11
T13	*Wataria parvipora*	33	TB9241	–c1	Supplementary Fig. 11
T14	*Wataria parvipora*	26	TB9242	–c1	Supplementary Fig. 11

Supplementary Table 2 Specimens of *Byttneriophyllum tiliifolium* and *Banisteriaecarpum giganteum* used in this study

Specimen number	Locality	Remarks
TB9100	Otb001	Supplementary Fig. 13b
TB9243	Site 3	Fig. 5d–f, Supplementary Fig. 13a
TB9244	Site 3	Fig. 5g
NSM-PP-23947	Hirakaido	Supplementary Fig. 14b
NSM-PP-23949	Hirakaido	Supplementary Fig. 14a
